# Trajectories and Milestones of Cortical and Subcortical Development of the Marmoset Brain From Infancy to Adulthood

**DOI:** 10.1093/cercor/bhy256

**Published:** 2018-10-11

**Authors:** S J Sawiak, Y Shiba, L Oikonomidis, C P Windle, A M Santangelo, H Grydeland, G Cockcroft, E T Bullmore, A C Roberts

**Affiliations:** 1 Behavioural and Clinical Neuroscience Institute, University of Cambridge, Downing Site, UK; 2 Wolfson Brain Imaging Centre, University of Cambridge, Box 65 Addenbrooke’s Hospital, Cambridge, UK; 3 Department of Physiology, Development and Neuroscience, University of Cambridge, Downing Street, Cambridge, UK; 4 Department of Psychiatry, University of Cambridge, Cambridge, UK; 5 Research Group for Lifespan Changes in Brain and Cognition, Department of Psychology, University of Oslo, Oslo, Norway; 6 ImmunoPsychiatry, GlaxoSmithKline Research and Development, Stevenage, UK

**Keywords:** myelination, spline trajectory, tensor-based morphometry

## Abstract

With increasing attention on the developmental causes of neuropsychiatric disorders, appropriate animal models are crucial to identifying causes and assessing potential interventions. The common marmoset is an ideal model as it has sophisticated social/emotional behavior, reaching adulthood within 2 years of birth. Magnetic resonance imaging was used in an accelerated longitudinal cohort (*n* = 41; aged 3–27 months; scanned 2–7 times over 2 years). Splines were used to model nonlinear trajectories of grey matter volume development in 53 cortical areas and 16 subcortical nuclei. Generally, volumes increased before puberty, peaked, and declined into adulthood. We identified 3 milestones of grey matter development: I) age at peak volume; II) age at onset of volume decline; and III) age at maximum rate of volume decline. These milestones differentiated growth trajectories of primary sensory/motor cortical areas from those of association cortex but also revealed distinct trajectories between association cortices. Cluster analysis of trajectories showed that prefrontal cortex was the most heterogenous of association regions, comprising areas with distinct milestones and developmental trajectories. These results highlight the potential of high-field structural MRI to define the dynamics of primate brain development and importantly to identify when specific prefrontal circuits may be most vulnerable to environmental impact.

## Introduction

Disorders of mental health affect up to half the population during their lifetime, but half of adult patients will already be diagnosed by age 14, rising to 75% by age 24. This is strongly suggestive of developmental factors in the pathogenesis of mental health disorders (Kessler et al. 2005; Keshavan et al. 2014). For example, disruption of the development of cortical and subcortical brain circuits underlying attention, decision making, mood and emotion likely contributes to early life incidence of psychiatric symptoms. Our current knowledge of the developmental trajectories of these circuits is, however, limited.

Advances in pediatric MRI have enabled studies of human brain growth over time (Giedd et al. 1996; Gogtay et al. 2004; Shaw et al. 2008; Raznahan et al. 2011; Khundrakpam et al. 2017) with a particular focus on adolescence where mental health disorders have their greatest incidence (Paus et al. 2008). These studies reveal distinct trajectories of growth, often emphasizing the progressive then regressive periods of grey matter volume and cortical thickness that mirror the cytoarchitecture and evolutionary development of the cerebral cortex (Shaw et al. 2008) (but, see Walhovd et al. (2017), for discussion of contradictions regarding the existence of a progressive period in studies of cortical thickness). In all studies, higher-order association regions of the prefrontal cortex, orbitofrontal cortex, and temporal lobes show later maturation compared with somatomotor regions of cortex. In contrast, white matter growth shows a steady increase well into adulthood. Given the long developmental window in humans spanning from birth to 21 years, the value of performing similar imaging studies in nonhuman primates with a more concentrated period of development is becoming recognized. Some progress has already been made in characterizing rhesus macaque (*Macaca mulatta*) brain development (Knickmeyer et al. 2010), with the most comprehensive study to date in 45 animals (Scott et al. 2016) which looked at developmental trajectories from 1 to 52 weeks across large subdivisions of the cerebrum (e.g., frontal, parietal, temporal, and occipital lobes) and subcortical regions including the brain stem. This study did not distinguish grey matter from white matter, but nevertheless identified the protracted growth of frontal and temporal lobes, consistent with human studies.

**Figure 1.**
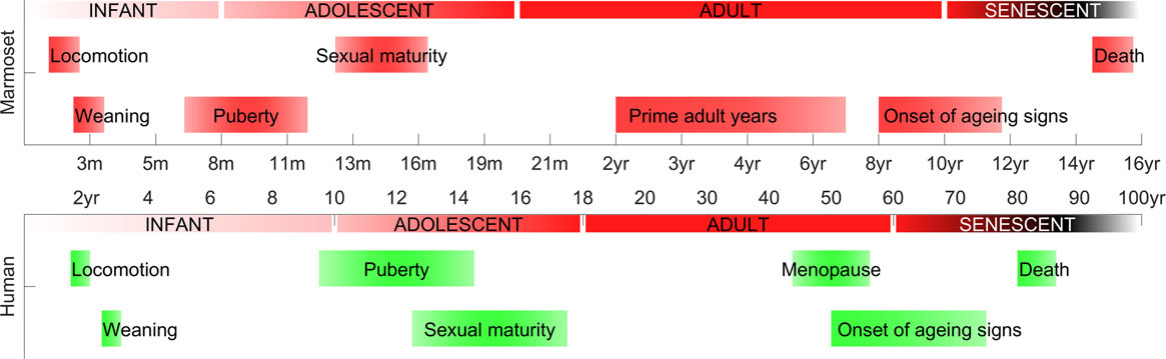
Developmental epochs in the life of the marmoset with broadly homologous periods in the human lifespan for comparison.

More recently, the suitability of the common marmoset, *Callithrix jacchus*, a small New World primate with a compact lifespan (10–15 years), has been recognized for studying all stages of development (Fig. 1) (Abbott et al. 2003; Schultz-Darken et al. 2016). Marmosets live in family groups, similar to humans, and exhibit human-like social behavior (Miller et al. 2016). They are an excellent primate species for neurobiological (Oikonomidis et al. 2017) and genetic studies (Kishi et al. 2014). Accordingly there have been a number of recent neuroimaging studies focusing on both prenatal and postnatal marmoset brain development (Hikishima et al. 2013; Seki et al. 2017) and histological studies of postnatal developmental changes (Bourne and Rosa 2006; Burman et al. 2007; Oga et al. 2013; Sasaki et al. 2015). Cortical grey matter volume in the marmoset brain demonstrates a similar inverted-U sequence of increase, plateau and decrease over developmental time as previously described for macaque and human brains.

Here we aimed to measure developmental changes in grey matter volume of the marmoset brain more comprehensively. We used spline models to quantify the nonlinear path of grey matter development at each of 53 neocortical areas defined by an anatomical template, and 16 major allocortical and subcortical structures, in each of 41 animals aged between 3 and 27 months. Each animal was scanned at least twice in an accelerated longitudinal design allowing us to model developmental changes from before puberty to early adulthood. We predicted that association cortex and subcortical nuclei would show a protracted time course of grey matter development compared with that of primary and secondary sensory and motor areas. Further, given that varied changes in prefrontal function and structure are associated with nearly all neuropsychiatric conditions, we hypothesized that prefrontal cortex may comprise a particularly heterogeneous collection of growth curves with distinctive developmental profiles.

## Materials and Methods

### Animals

All procedures were in accordance with the UK Animals (Scientific Procedures) Act 1986 under licence PPL70/7618 and approved by the University of Cambridge Animal Welfare and Ethical Review Board.

The design adopted was a compromise between a pure longitudinal design and a cross-sectional design. In designing a study of this type, there must be a trade off between focussing heavily on particular individuals or scanning widely across a population. The longitudinal assessment of individuals is crucial for unmasking details of the developmental trajectories. However, by including data from many individuals we have been able to additionally capture the variability in the population more widely. In this way, our design captures trajectory details that are more likely to generalize to the population. Images were obtained from 3 to 27 months at quarterly intervals. Cohorts of a minimum of 15 animals were obtained between 3 and 21 months.

Marmosets were all born and reared in the University of Cambridge marmoset colony. Animals were housed in family groups in rooms with controlled humidity (50%) and temperature (21 °C), with water available *ad libitum*. They were fed peanuts or Farley’s rusk in the morning and sandwiches (wholemeal bread, marmoset jelly [Special Diet Services, Essex, UK] hardboiled egg), and 2 pieces of fruit in the evening. Besides MRI scans as detailed below, marmosets in this study did not undergo any other experimental procedures while part of this study.

On the day of scanning, animals were fasted. Marmosets were sedated with ketamine (20 mg/kg; Vetalar solution 100 mg/mL Pfizer, Kent, UK) and intubated for isoflurane anesthesia (2.5% in 0.25–0.40 L/min O_2_). Throughout scanning, respiration rates were monitored with a pressure sensor over the upper abdomen (SA Instruments, Stony Brook, NY) and the isoflurane dose was adjusted between 1% and 3% to maintain respiratory rates in the normal range. A rectal thermometer was used to monitor temperature and a flowing-water heating system was adjusted as necessary. Marmosets were returned to their home cages once recovered.

Marmosets were regularly assessed for health, including monthly weighing, as part of the normal colony procedures. No adverse health events were recorded for any of the animals in this study.

We scanned 41 animals a total of 147 times with an average of 3.7 scans per animal (range 1–7 scans; *n* = 38, 33, 19, 10, 45, 1 having ≥2, 3, 4, 5, 6, 7 scans, respectively).

### Image Acquisition and Processing

Animals were scanned using a Bruker PharmaScan 47/16 MRI system (Bruker, Inc., Ettlingen, Germany) at a field strength of 4.7 T. A custom 6 cm birdcage coil was used for signal transmission and reception. Structural images were acquired using a rapid acquisition with relaxation enhancement (RARE) sequence (parameters: TR/TEeff 11 750/23.5 ms, 125 slices of 250 μm thickness, echo train length 4 and 3 averages) in 21 m 44 s. The field of view was 64 mm × 50 mm yielding an isotropic resolution of 250 μm.

Images were processed using SPM8 (Wellcome Trust Center for Neuroimaging, UCL, UK) with the SPMMouse toolbox for animal data (Sawiak et al. 2013). Brains were aligned with tissue probability maps derived from marmosets from the same colony (Mikheenko et al. 2015) and segmented into grey matter, white matter, and cerebrospinal fluid (GM, WM, and CSF). DARTEL (Ashburner 2007) was used for image registration and to create population templates. Tensor-based morphometry (TBM) was used to find volume differences at 3 stages: “prepubertal” (changes from 3 to 6 months), “pubertal” (between 6 and 12 months), and “postpubertal” (between 12–21 months). Jacobian determinant maps for each scan were produced from the DARTEL flow fields and smoothed with an 800 μm isotropic Gaussian smoothing kernel. A general linear model was used with a block design for ages at 3-month intervals, including covariates for sex, subjects (i.e., a random effect for each marmoset), and total intracranial volume (TIV) so as to remove changes that can be explained by overall brain volume. Additionally, to assess sex differences, a block design was used to compare male and female animals within each age block.

Mean warped images were calculated from all subjects and time points, in addition to separate average images made from brain scans in 3-month intervals from 3 to 21 months. These quarterly maps were segmented and registered with DARTEL to create deformation fields between them so that a movie could be made to simulate a continuous transition of brain structures from infancy to adulthood (Supplementary Movie 1).

With DARTEL, linear elastic regularization was used with the default parameters. Smoothing parameters were adjusted for the marmoset brain (for the 6 iterations: 0.034, 0.017, 0.008, 0.004, 0.002, and 0.001).

An adjusted *P*-value was used to control the type I error rate expected due to multiple comparisons, calculated for a false-discovery rate (Genovese et al. 2002) of *q* < 0.05. Two-tailed tests were calculated in each case.

### Cortical Parcellation of Brain Regions

Cortical brain regions were defined by adapting a digital reference template based on histological sections of the marmoset brain (Majka et al. 2016), itself based on a comprehensive atlas (Paxinos et al. 2012). The unilateral labels of this dataset were flipped symmetrically about the midline to produce a symmetric atlas of the entire cortex. The cortex of the structural (DARTEL) MRI template derived from all the scanned brains in this study was manually delineated as a whole and image registration was used to find the best affine mapping to a mask corresponding to the histologically labeled data. Each voxel of the MRI cortex mask was then assigned to the nearest corresponding labeled voxel from the histological dataset.

The original histological atlas comprised 116 regions, many of which were distinguished on the basis of detailed cytoarchitecture or functional differences that are not evident in the MRI data. Particularly for small, ill-defined (in terms of MRI contrast) areas, including this level of detail would be unwarranted. For this reason, the original histological atlas was simplified such that subdivisions of Brodmann areas were not considered separately. A complete list of regions is given in Supplementary Table S1.

The cerebellum, subcortical and allocortical structures including the head (ventromedial and dorsolateral nuclei) and body of the caudate, putamen, nucleus accumbens, amygdala (basolateral and central nuclei), medial thalamic nucleus, septum, hypothalamus, and the anterior aspects of the hippocampus were delineated using Analyze 8 (Mayo Clinic) by an expert reviewer (ACR) with reference to a histological atlas (Paxinos et al. 2012). Additional structures segmented included the habenula and raphe nuclei (dorsal and medial).

### Regional Growth Trajectory Analysis

The volumes of 69 symmetric cortical, subcortical, and allocortical regions and 3 regions across the midline were estimated by integrating Jacobian determinants from the DARTEL transformations over each region mask. No filtering based on tissue segmentation was used in this process. An additive mixed model was used to model these volumes using cubic splines (Alexander-Bloch et al. 2014) with all computations performed in Matlab 2016b (Mathworks, Inc.). The volume of the ith structure in the jth subject, $V_{i,j}$ at age t is modeled as follows:

(1) $$V_{i,j}\left(t\right)=\beta_{s}S_{i}+f_{i}\left(t\right)+u_{i,j}$$

where $S_{i}=1$ for female subjects and 0 for male subjects and $f_{i}\left(t\right)$ is the same function of age for all subjects, giving the fitted spline for each structure. $u_{i,j}$ is the random effect of subject (here an additive constant: varying by structure and subject but not with time).

**Figure 2.**
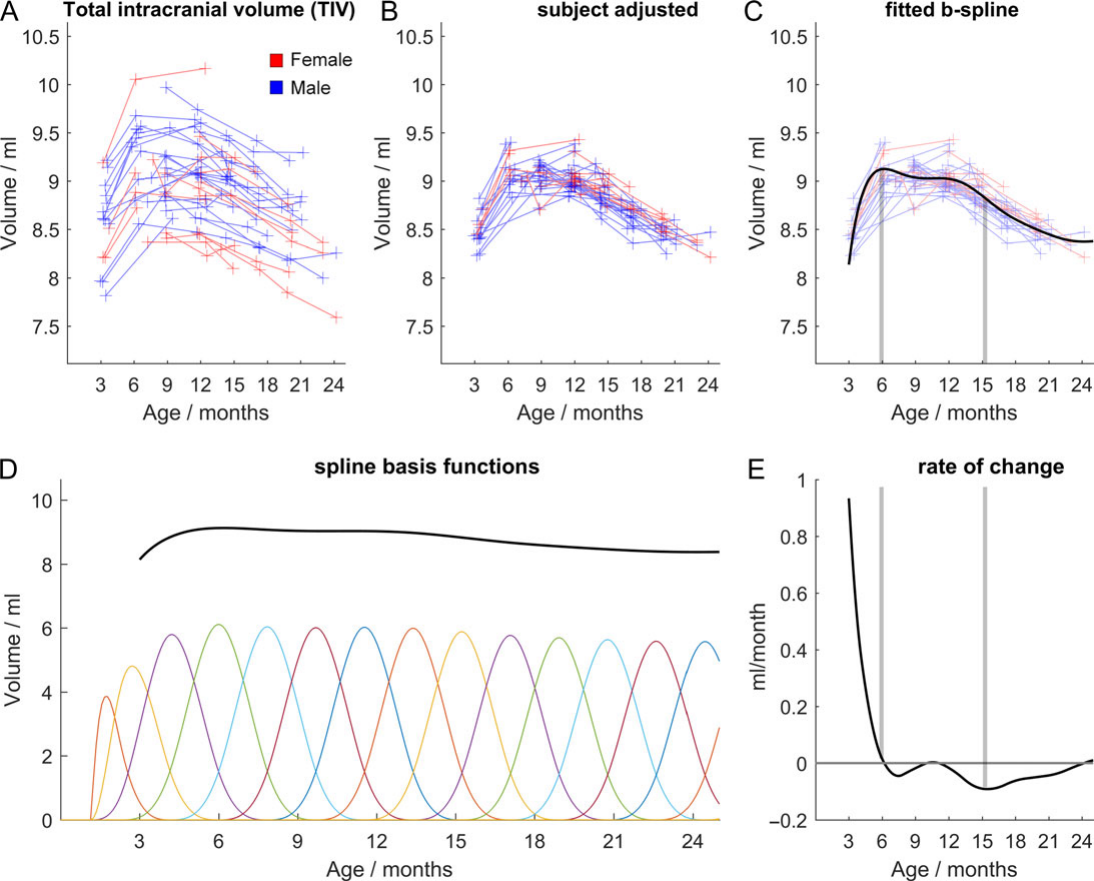
Growth trajectory modeling using total intracranial volume (TIV) for illustrative purposes. The effect of subject is modeled as an additive constant to the growth trajectory which is fitted with B splines. Raw data (*A*) is shown with subject effects (i.e., the mean of each subject trajectory) removed (*B*) and the fitted spline in (*C*). The basis functions for this curve are shown in (*D*), each is adjusted to best fit the data. As each adjustment is local, it only has an effect in its vicinity or “knot.” The spline derivative is shown in (*E*), highlighting the rate of change of TIV. Vertical lines shown in (*C*) and (*E*) indicate how a zero in the derivative corresponds to a maximum volume (6 months) and how the minimum of the derivative curve (at 15 months) coincides with the greatest rate of volume loss in (*C*). The functions used here were based on a penalized spline calculated with a constant smoothing factor. Gradient descent with linear least squares minimization was used to fit the additive model, iteratively updating subject offsets and spline coefficients to minimize the squared errors.

The process of fitting this model is illustrated in Figure 2. The spline fits are based on the sum of cubic b-splines, a set of smooth local basis functions each of which is adjusted to best fit the data. Each adjusted function only affects the fit in the vicinity of its time point, or “knot.” The functions used here were penalized splines with a constant smoothing factor (Shaw et al. 2008). Following (Alexander-Bloch et al. 2014) we used 10 knots over the age range of interest (3–24 months). Further knots with the same spacing were included outside of this range to avoid spurious edge effects.

Based on the inverted-U shape of these trajectories, we defined 3 key milestones. All of the regions show growth from infancy, reaching a peak as either a sharp, well-defined maximum or a longer plateau, before the volume starts to decrease. The 3 milestones capturing this feature were defined as follows: 1) the age at peak volume, 2) the age at onset of volume decline, and 3) the age at maximum rate of volume decline. Milestones were estimated using the first derivative of the spline grey matter volume change in each region. At the peak, the first derivative is zero. By bootstrap resampling, 95% confidence intervals were obtained for the age at which the derivative was not zero. The age of onset of decline was defined as the upper limit of that interval. Finally, the age at maximum rate of decline coincides with the time of the minimum of the first derivative curve for each structure. To maintain the nested structure of the data, bootstrap resampling was performed over scans, preserving identity information to allow individual subject offsets to be taken into account for the analysis.

### Trajectory Clustering

We used cluster analysis to subdivide the marmoset cortex into a few large systems or families of cortical areas sharing a distinctively similar developmental profile. At each of the first few levels of this neurodevelopmental hierarchy we found corresponding *k*-means clustering solutions with k in the range 2–10. These could be compared with each other in terms of the cluster-wise mean volume trajectory and also in terms of the 3 milestones of cortical volume change.

Briefly, the *k*-means method randomly allocates feature vectors from each region (in this case, the vector of spline coefficients defining each areal trajectory) to one of *k* distinct clusters, which are subsequently updated as the mean of all their allocated regions. Each region is then reassigned to its nearest cluster and the process repeated until it converges. All computations were performed using the algorithms of the Statistics and Machine Learning Toolbox with Matlab 2016b (Mathworks, Inc.).

There is no consensus on how to determine an “optimal” number of clusters to describe complex datasets and we therefore explored results over a range of values of *k*. We focused on *k* = 6 because this was the largest number of clusters for which each cluster was significantly different from all other clusters in terms of at least 1 developmental milestone (see Supplementary Fig. S2).

### Cerebral Asymmetry

To assess cerebral asymmetry, trajectories were computed for left and right hemisphere ROIs independently, using equation 1 as before.

Further, for a voxel-based analysis, the population templates produced by DARTEL were processed by reflecting the data in the left–right direction, using a rigid registration (computed by SPM8) to align the original and reflected templates, and averaging them together resulting in a symmetric template. Each scan was then warped to match the symmetric target with DARTEL to produce Jacobian determinants (i.e., volume change) maps. Left–right differences were computed by subtracting a reflected copy from each map such that positive values would indicate larger volumes compared with the corresponding contralateral region. These asymmetry maps were tested with a 2-tailed *t*-test (*P* < 0.05 FDR-corrected, as for the tensor-based morphometry analysis) at 3, 12, and 21 months.

## Results

**Figure 3:**
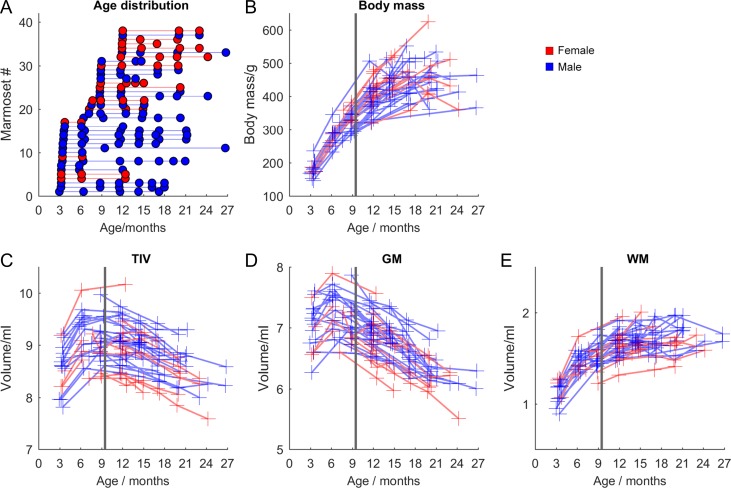
(*A*) Ages of each marmoset scanned. (*B*) Total body mass. (*C*) Intracranial volume. (*D*) Total grey matter volume. (*E*) Total white matter volume. The solid vertical line marks the mean age of puberty in the animals for reference.

**Figure 4.**
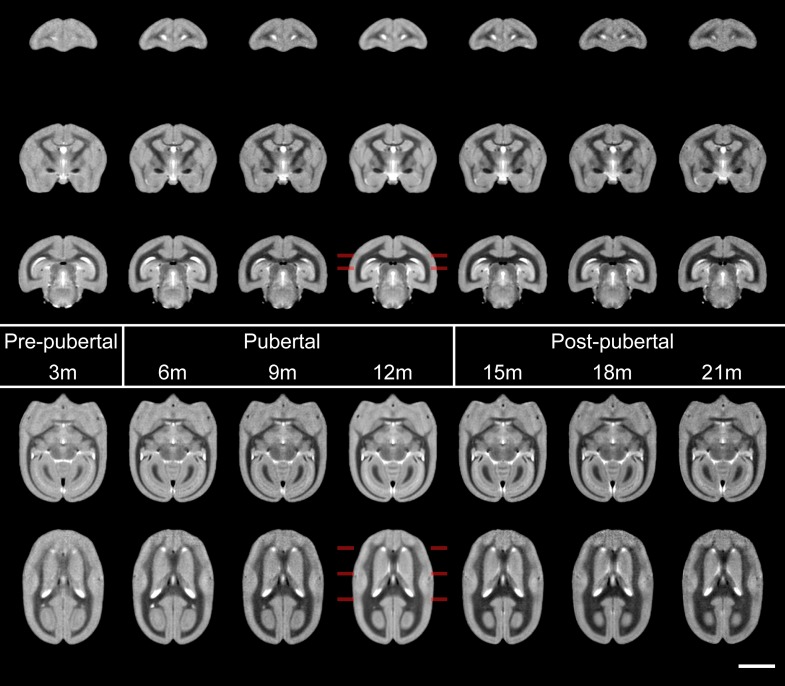
Sections from mean templates of animals produced at regular intervals from 3 to 21 months, from prepuberty, through puberty and postpubertal stages from left to right. With the imaging parameters chosen, WM yields the darkest intensity, followed by GM and then CSF yields the brightest. Changes in WM are apparent at each age point: not only does it appear markedly thicker, but darker with time. Visually it appears to increase in volume at the expense of surrounding GM. Marked increases in white matter include that within the prefrontal cortex (coronal sections: top row), internal capsule (middle row 2) and corpus callosum (bottom row) and the anterior commissure (horizontal sessions: top row) and visual cortices (bottom row). Changes with age are readily appreciated visually with the simulated development movie (Supplementary Movie 1). Coronal sections are taken at 14, 6, and -2 mm from the intra-aural line, horizontal sections at 0 and 3.5 mm from the AC–PC line, indicated at the 12 m template. Scale bar is 5 mm.

**Figure 5.**
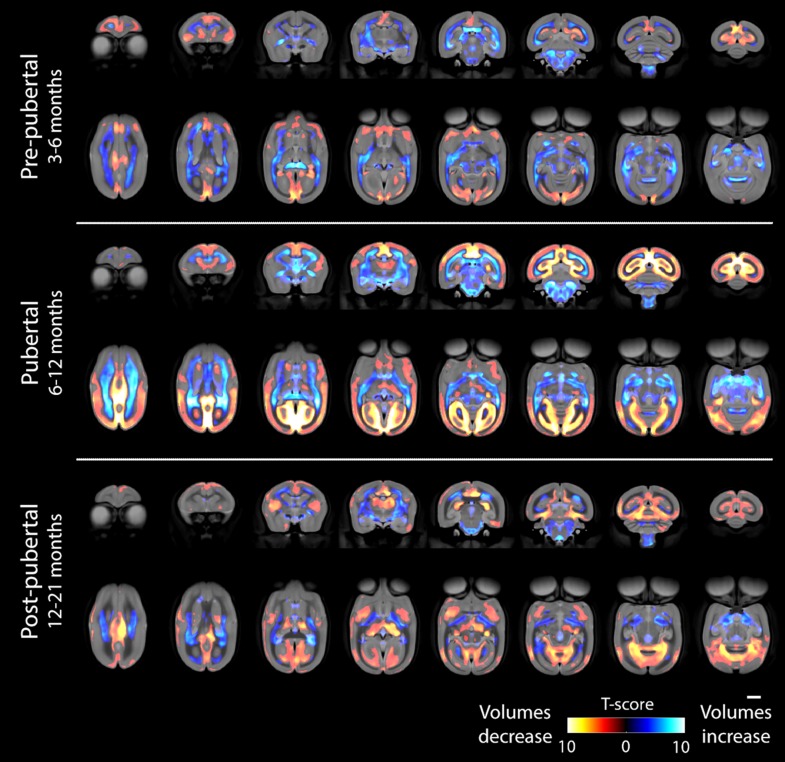
Tensor-based morphometry results showing regions where volume increases or decreases between different stages are not explained by TIV changes. The color bar shows Student’s *T*-score and all changes shown are significant at an adjusted threshold for *P* < 0.05 (false-discovery rate; 2-tailed). The scale bar shows 5 mm. The top row of each timepoint shows coronal sections (slices progressing anterior to posterior) and the second row shows axial sections (superior to inferior).

The body mass of animals measured at each scan is shown in Figure 3, approximately doubling (+122%, *P* = 1 × 10^−21^; 95% CI: 108–136%) pre-to-post puberty (3–12 months), approaching a plateau (a further increase of 11% is seen from 12 to 15 months, *P* = 0.005, 95% CI: 4–19%) before stabilizing from 12 to 24 months. Brain volumes do not share the same trajectory (Fig. 3*C*–*E*). TIV (here calculated as the sum of segmented GM, WM, and CSF portions) increases by 7% in the prepubertal stage (3–6 months, *P* = 3.8 × 10^−4^, 95% CI: 3.4–10.6%), with total GM volume increasing by 3.7% (*P* = 0.05, 95% CI 0.1–7%) and total WM volume increasing by 28% (*P* = 1 × 10^−9^, 95% CI: 21–35%). During puberty (6–12 months), GM volume falls by 5% (*P* = 0.001, 95% CI: 2–8%) but WM volume increases by 11% (*P* = 7 × 10^−5^, 95 CI% 6–16%). Postpuberty (12–21 months), WM volume appears to plateau (increasing 4%, *P* = 0.2, 95% CI: −2% to 10%), whereas GM volume falls by a further 9% (*P* = 7 × 10^−8^, 95% CI: 6–11%). TIV in the same period decreases by 6% (*P* = 6 × 10^−4^, 95% CI: 3–9%). Further age-related changes in GM/WM volumes were evident by inspection of the average image at each of the seven 3-month epochs from 3–6 to 21–24 months (Figs 4 and 5 and Supplementary Movie 1). WM grows and its signal becomes more distinct over time, with complementary attenuation or shrinkage of anatomically adjacent GM signals in cortex and subcortical nuclei.

We used tensor based morphometry (TBM) to assess the statistical significance of age-related changes in grey and white matter between the 3 major phases of prepuberty (3–6 months), puberty (6–12 months), and postpuberty (12–21 months) (Fig. 5). Areas of volume growth and reduction are shown with different color scales. In each map, relative volume reductions are virtually all in grey matter regions, with corresponding increases in white matter regions. Prepuberty, GM changes are particularly striking in the frontal cortex, insular cortex, cingulate cortex, and medial aspects of primary visual cortex. During this stage of development, major white matter tracts change substantially, most clearly in the anterior commissure, at the splenium of the corpus callosum and in the inferior longitudinal fasciculus. Except for the corpus callosum, it is generally WM in more inferior parts of the forebrain that shows significant change during this time period.

Later, during puberty, the differences are much more significant throughout the brain. Now, prefrontal cortex shows less change, but sensory and motor cortices show significant reductions in GM. Occipital GM has the most substantial volume loss, with the entirety of primary visual cortex V1 and V2 showing marked changes in this period (these differences are also clear from visual inspection of Fig. 4). In terms of WM, tracts thickening in the prepubertal period continue to do so but are joined by the superior longitudinal fasciculus and the external capsule. The corticospinal tracts and midbrain WM also increase in size.

Postpuberty, the trend of decreasing GM in favor of increasing WM continues. Subcortical GM is seen to change prominently for the first time, most clearly in the putamen. Anterior and posterior cingulate cortex, particularly in the inferior aspects, continue to undergo significant GM loss but more lateral regions of the parietal and occipital cortices show fewer differences in this period. As during puberty, medial regions of visual cortex continue to change but at this point it is more focussed within the inferior aspects.

No significant sex differences were detected at any age point in total body mass, GM, WM, or TIV; nor using TBM within each age block. This is consistent with previous studies in marmosets (Sakai T et al. 2017), but diverges from differences reported in humans (Giedd et al. 1996). It may be that more subtle findings could exist and be detected with a larger cohort at all age points. It is not straightforward to sex marmosets at 3 months and unfortunately we had only 4 of 17 female marmosets at this age. Looking specifically at 12 months, however, in a comparison of 12 female versus 19 male animals there were no significant volume differences seen with TBM. These are reasonable group sizes for comparison, implying that any sexual dimorphism in brain volume that does exist is relatively minor. We therefore only report results for pooled (male and female) data.

Bootstrap confidence intervals for left and right hemispheres overlapped at every time point for every ROI tested, indicating no significant asymmetry in the brain visible at an ROI level. In the voxel-based analysis, a small yet consistently significant region of the posterior inferior aspects of hippocampus showed a greater volume on the right at 3, 12, and 21 months (Supplementary Fig. S1) as did the left Sylvian fissure. A longer left Sylvian fissure, compared with the right, was found in the brains of 4 of 5 species of New World monkey (including marmosets) studied at post mortem by (Heilbroner and Holloway 1988). However, this finding was not reproduced in an MRI study of 2 of these species by (Pilcher et al. 2001), which incidentally does provide a useful survey of laterality studies and their conflicting results in nonhuman primates. The only other areas of asymmetry were found in the dorsolateral putamen at 12 and 21 months and the ventrolateral and ventroposterior thalamus at 21 months only.

### Trajectory Analysis

**Figure 6.**
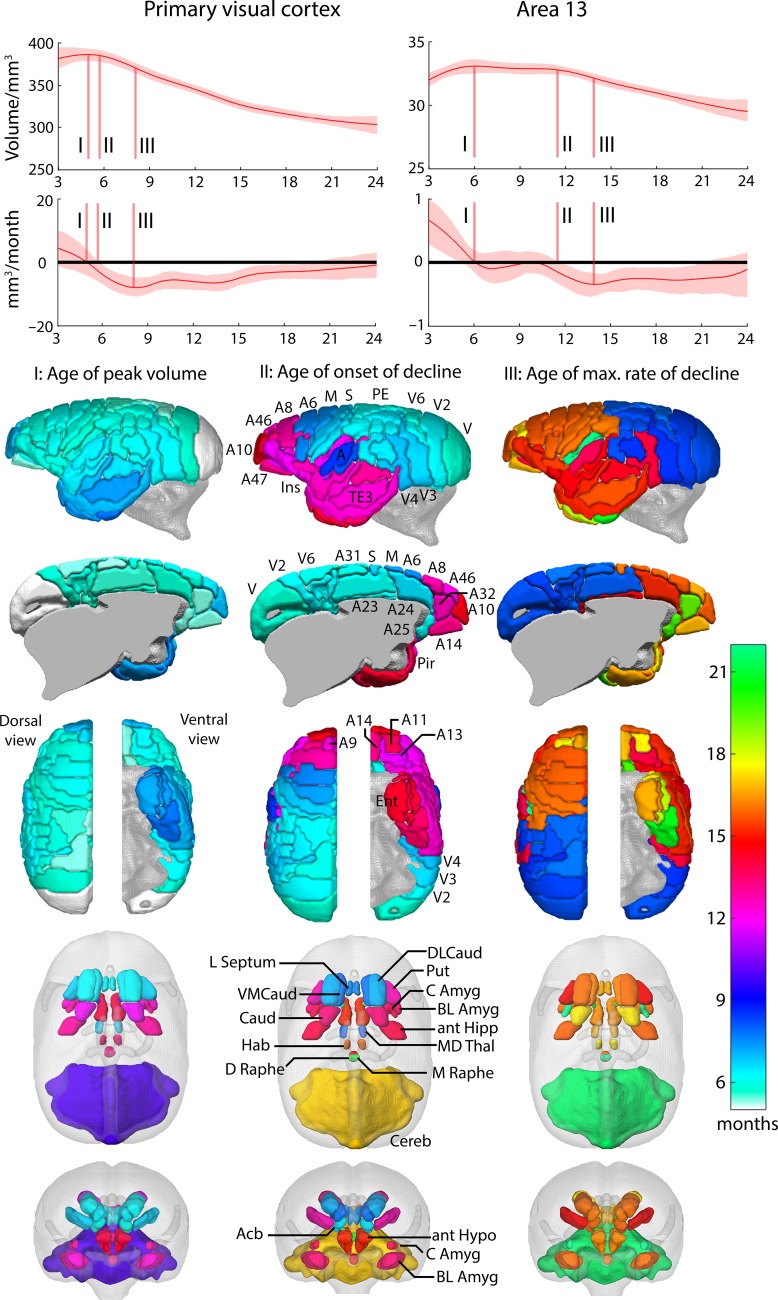
Growth trajectories from fitted splines for primary visual cortex and orbitofrontal area 13 to illustrate milestones of development. The shaded region shows the 95% bootstrap confidence interval for the curve. The initial growth peak I) is the first maximum seen and the plateau end II) occurs when the 95% confidence interval for the rate of change no longer includes zero. The maximum rate of change following the growth peak defines stage III). These features can be seen most clearly in the first derivative curves, which display the rate of change of volume as a function of age. The first difference of note in the growth trajectories is the time at which each region reaches its greatest volume (milestone I); 4.98 months for V1 and 6 months for area 13. In V1, there is only a short period of approximately 1 month, before the onset of volume decline at 6 months (milestone II). In contrast, the peak volume of area 13 remains unchanged for approximately 6 months before starting to decline at 12 months. 3D reconstructions show the age at which these features occur for each cortical and subcortical region.

Inspection of the growth trajectories revealed a number of features that differentiate cortical regions during development (Fig. 6). For illustrative purposes, the fitted curves of the splines and their derivatives are shown for a primary sensory area, V1, and a higher association area, 13, in prefrontal cortex. A complete set of trajectories for every brain region is provided in Supplementary Material. Brain maps of these key developmental milestones highlight marked differences within cortical lobes as well as between cortical and subcortical regions (Fig. 6). The initial peak volume (I) for all cortical structures is just prior to, or during the early stages of puberty (5–7 months). This feature discriminates between primary and association cortex, particularly within visual cortex, with V1 peaking first (month 5), and a trend to later peaks for V3, V4, and V5 (month 6), but not so for V6 (month 5.4), consistent with the view that this area is distinct from V3 (Angelucci and Rosa 2015). Primary motor and somatosensory cortices peak shortly after V1, months 6.0 and 5.9, respectively, whilst primary auditory cortex is somewhat delayed: reaching its peak along with the auditory belt and parabelt regions at month 6.4. Later still are those regions within the lateral and inferior temporal lobe and the temporal and frontal poles, not reaching their peak until 7 months.

An even greater difference is seen between cortical brain regions with respect to the age of onset of the decline in cortical volume (II). Much of the posterior sensory regions and primary motor and premotor (A6) regions begin declining between 6 and 8 months during puberty. In contrast, the majority of the prefrontal cortex, including orbitofrontal and perigenual cingulate cortex (area 32), and much of the anterior temporal lobes, start to decline much later, around 12–14 months, after puberty has finished. There are noticeable exceptions though. Cingulate association regions including subgenual area 25, dorsal anterior area 24 and posterior area 23 have an onset of volume decline much earlier (months 6–6.5), closer to that seen in posterior sensory cortices and motor cortex.

The division between cortical brain regions in the age at which they display the maximum rate of decline in volume (III) varies considerably from the divisions seen in the age of the onset of decline (II). Consistent with the latter is the relatively early age, before or at the beginning of puberty (7.9–8.5 months), that parietal and occipital regions display their fastest rate of decline compared with that of prefronto-temporal areas at the end of puberty and sexual maturity (14–20.7 months). However, other brain regions that exhibited a relatively early age of onset of volume decline including primary and secondary somatosensory (S2) and premotor (area 6)/motor (area 4) regions (6.6–7.4 months) showed their fastest rate of decline much later (15.9–22 months) like that of prefronto-temporal areas. This was also the case for area 25 which showed one of the earliest onsets of decline at 6.0 months but one of the latest time-points for maximum decline (19.8 months), alongside area 32 (19.4 months), secondary somatosensory (22 months), and the ventral temporal lobes (20.8 months). This suggests that the period of volume decline is particularly prolonged in area 25.

Many of the subcortical regions studied here (nucleus accumbens [Acb], putamen [Put], dorsolateral and ventromedical caudate [DLCaud/VMCaud], anterior hippocampus [antHIPP], lateral septum [Lseptum], and mediodorsal thalamus [MD Thal]) reach peak volume before puberty (5.8–6.9 months), similar to cortical regions. However, this is not the case for the anterior hypothalamus, basolateral and central amygdala, basal nucleus of the stria terminalis, habenula, dorsal and medial raphe nucleus that peak much later, between 10 and 16 months. Of those that peak early, only Acb, DLCaud, VLCaud, Lseptum, and MD Thal also show an early onset of decline (6.5–8.0 months). The rest, along with those subcortical regions that peaked much later, showed an onset of decline between 13.4 and 21 months. Of note was the marked difference in the onset of decline between striatal regions, with Acb, DLCaud and VLCaud declining at 6.5–7.8 months compared with the Put and Caud declining between 12.8 and 13.4 months. The age of maximum rate of decline for all subcortical structures is postpubertal (in general 15–17 months), but as late as 22–23 months, in early adulthood, for AmygCE and cerebellum.

### Clustering of Neocortical Regions

Clustering provided a more data-driven technique to identify *k* > 2 families of cortical areas that shared a distinctive developmental profile. This provided an alternative approach to describing the data. The clustering was based on the whole trajectory in order to reveal those cortical regions with overall similar profiles. In addition, we assessed how well the 3 milestones that we had calculated from each trajectory were representative of the whole. Moreover, comparison of pairs of milestones at the level of clusters emphasized distinctions between groups of cortical regions sharing distinct developmental features.

**Figure 7.**
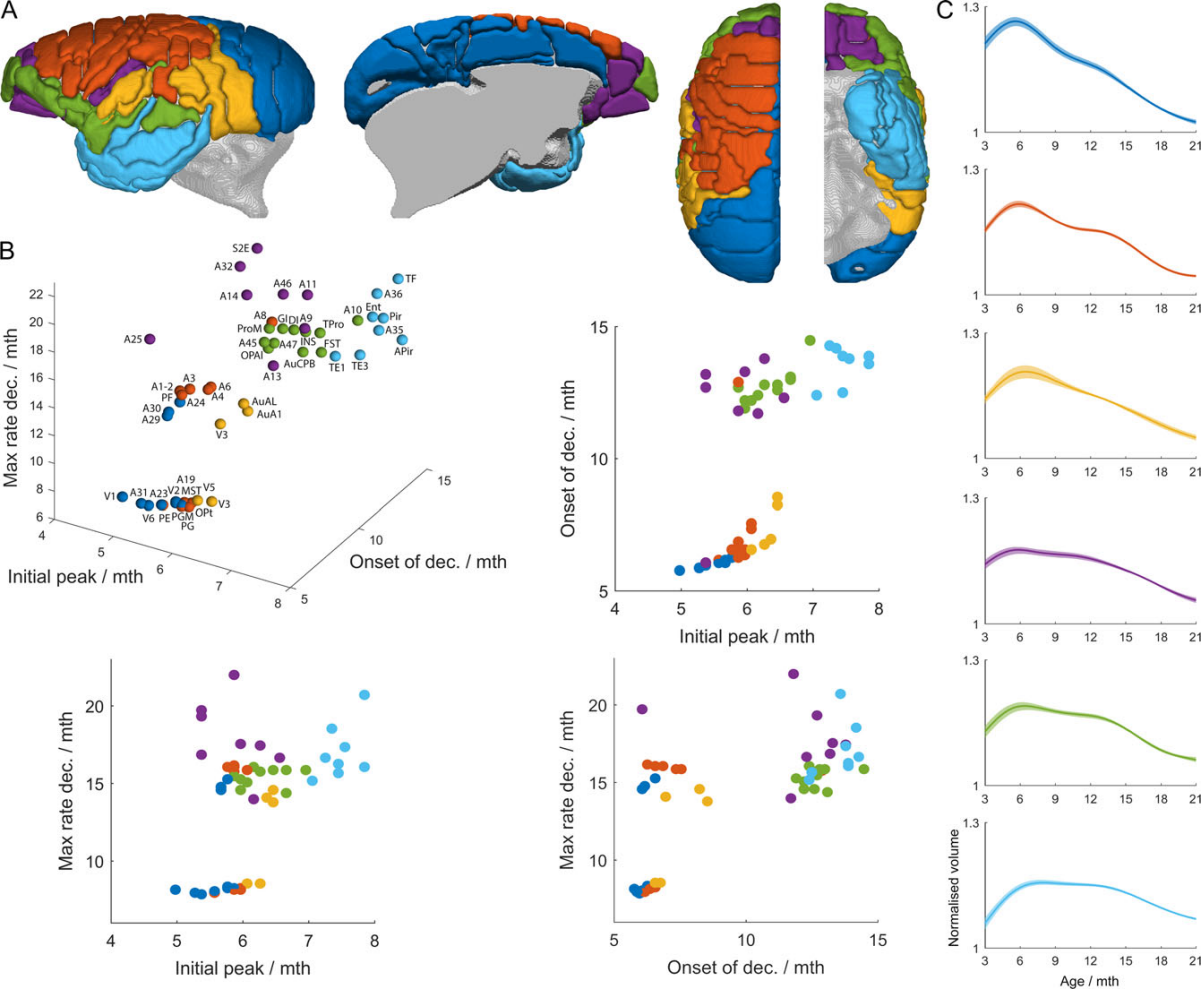
Cortical clustering by trajectory. (*A*) shows 6 clusters found by *k*-means clustering. (*B*) The clusters are well-differentiated when considered in a 3D space formed by the milestone markers of Figure 6: the age of peak grey matter volume, the age at onset of grey matter volume decline, and the age at which the volume loss is a maximum. (*C*) the mean trajectory for each cluster is shown, with the shaded region indicating a 95% bootstrap confidence interval.

We focused on *k *= 6 as the solution representing the largest number of developmental families that were significantly distinct from each other (Supplementary Fig. S2). Figure 7 illustrates the differences in the profiles of the average trajectories of each of the 6 clusters. When taking into account all 3 milestones (3D plot in Fig. 7*B*) it can be seen that the clusters represented by the 6 distinct colors do form relatively separate groupings and ultimately these 3 milestones do capture the key elements of the trajectories representing the 6 clusters shown in Figure 7*C*. By comparing pairs of milestones in relation to these clusters (2D plots in Fig. 7*B*) a bimodal split in the age of onset and maximum rate of decline can be clearly seen. For the visual and cingulate cluster (1), somatomotor cluster (2), and auditory–visual cluster (3) onset of decline occurs at, or before the onset of puberty, soon after the point of peak volume. In contrast, onset of decline occurs at a much later timepoint after the point of peak volume, post puberty, for the orbitofrontal, dorsolateral, and ventromedial prefrontal cluster (4), ventrolateral PFC, polar, operculum, and insula cluster (5) and the lateral and inferior temporal lobe cluster (6). In addition, in the 2 clusters (4 and 5) involving prefrontal cortex, there appear to be 2 phases to the decline, an early shallow volume decline followed by a later more pronounced volume decline (maximum rate of volume decline).

**Figure 8.**
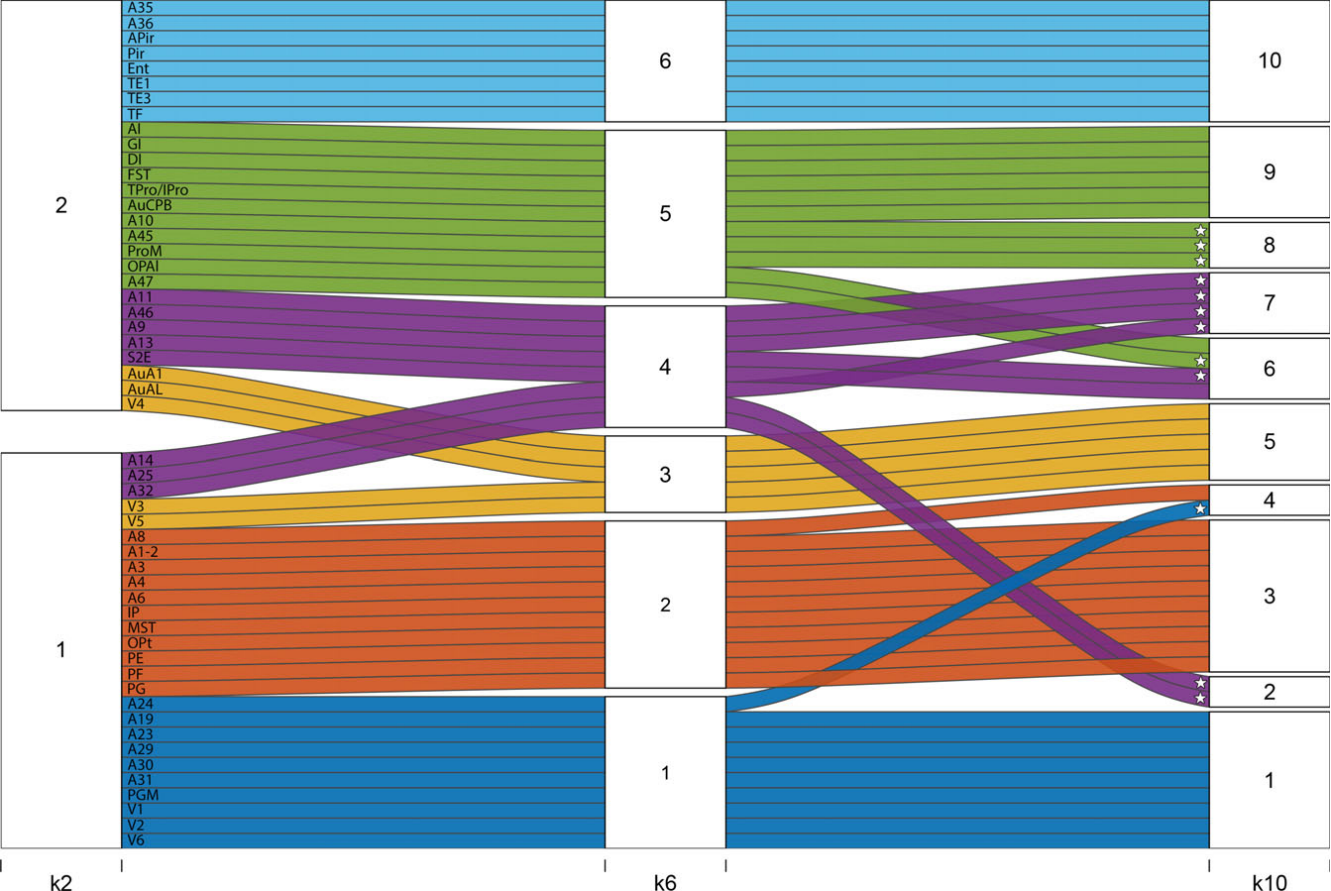
Alluvial plot showing how clusters evolve from *k* = 2 to *k* = 6 and *k* = 10. At *k* = 2, the cortex is subdivided into one large predominantly prefronto-temporal family of areas (cluster 1: areas which have relatively delayed onset of peak growth, a more prolonged plateau period before the onset of volume decline, and a later age at maximum volume loss), and one large predominantly occipito-sensorimotor-parietal family of areas (cluster 2). Greatest heterogeneity in terms of distribution across clusters is seen among the prefrontal cortical structures (marked with *). Coloring depicts the *k* = 6 cluster solution. Pale blue (6): lateral and inferior temporal lobe. Green (5): ventrolateral PFC, polar, operculum, and insula cortices. Purple (4): orbitofrontal, dorsolateral and ventromedial prefrontal cortices. Yellow (3): auditory‐visual. Orange (2): somatomotor. Dark blue (1): visual and cingulate cortices: Note that the 2 prefrontal clusters (green, 5 and purple, 4) split into 3 additional clusters between *k *= 6 and *k* = 10 such that at *k* = 10 prefrontal/cingulate regions contribute to clusters 2, 4, 6, 7, and 8.

Since there is no consensus for a definitive choice of *k* we also show the hierarchical relationship of *k* = 6 to those of *k* = 2 and *k* = 10 in Figure 8. Of particular note is that clusters from *k* = 7–10 involved further fractionation of the prefrontal cortex, which showed the greatest heterogeneity of growth curves compared with all other association regions.

## Discussion

Detailed analysis of growth trajectories across 69 cortical and subcortical regions in marmosets from 3 to 27 months, using an accelerated longitudinal design, revealed 3 major features of grey matter development. These milestones of development were age at peak volume, onset of volume decline, and maximum rate of volume decline. Comparison of the resulting age maps showed that whilst cortical regions differed in the timing of peak volume by just 3 months (months 5–7), the period in which the volume began to decline was much more protracted, ranging from 6 to 14 months, with the maximum rate of decline occurring between 10 and 20 months. These feature-specific age maps revealed unique differences in trajectories within and between cortical and subcortical regions that varied between features. The prefrontal cortex was the most heterogenous of all the association regions with multiple areas displaying distinct milestones and developmental trajectories.

### Developmental Neuroimaging of Primate Brains

To date, developmental neuroimaging studies in humans and nonhuman primates have revealed a number of key features of cortical development. There is considerable cerebral growth from infancy to adolescence, being greatest in humans and chimpanzees (both ~30%), but considerably lower in macaques (11%) (Sakai et al. 2013) and marmosets, as seen here (7%). Although GM increases before puberty are not consistently reported in humans (Walhovd et al. 2017), there is broad agreement that GM declines during development, whereas WM increases throughout infancy and puberty and post pubertal adolescence. This has been shown in humans (Giedd et al. 1996; Gogtay et al. 2004; Shaw et al. 2008) and more recently marmosets (Seki et al. 2017); and, in both species, differences in the timing of peak GM volume and rate of decline distinguish cortical brain regions. This overall pattern is also seen in the present study. In contrast, the decline in GM has not been seen so clearly in studies of the macaque monkey. In the 2 studies that followed development across a long enough developmental time period, one study (Scott et al. 2016) did not distinguish between grey and white matter and so the continuing rise in volume that they reported most likely reflects the continuing rise in white matter that has been reported in other studies. In the other study (Knickmeyer et al. 2010) only a rise and subsequent decline was seen in the prefrontal region. All other cortical regions showed either a continuing rise or no change. As that study was purely cross-sectional, it is likely that individual variability masked details of the developmental trajectory exposed in a longitudinal design. Individual variability can be substantial (compare panels *A* and *B* of Fig. 2 to see the effect of allowing for individual variability in elucidating growth trajectories).

In studies of macaques and previous studies of marmosets there was relatively gross parcellation of the cortex, primarily at the level of cortical lobes (e.g., parietal, occipital). In contrast, in humans, when a more detailed analysis is performed at the level of specific cortical regions, substantially different developmental trajectories have been identified that differentiate isocortex (complex cubic trajectory) from older allocortex (linear or quadratic trajectories) (Shaw et al. 2008).

### Growth Modeling and Milestones

The current results provide important new insight by characterizing 2 additional features of the developmental growth trajectories that, combined with an even finer-grained analysis at the level of individual cytoarchitectonic divisions, have revealed a more complex array of developmental time windows. Hence, they provide greater differentiation of primate cortical growth patterns than hitherto described. Importantly, they not only replicate previous findings that the frontal and temporal cortices develop later than all other cortical regions but they reveal that the variability in the timing of development within prefrontal cortex is the most heterogeneous of all cortical regions.

The first of these features is the plateau length. The second is the age at which the maximum rate of decline occurs. Previous modeling of cortical growth curves has not had sufficient degrees of freedom to capture these additional features. Together with maximum volume, these 3 developmental milestones reflected in the growth trajectories of the GM most likely represent distinct ages at which numerous different physiological/cellular processes are occurring. The increase and decline (inverted “U” shape) in structural volume measurements is a direct consequence of not only brain shape and localized volume changes, but changes of the MRI signal itself. This can occur because of changes in the fraction of watery “cytoplasmic material,” like cell bodies, synapses or extracellular fluid. Thus, the early increases in GM volume reported here likely reflect the critical periods of experience dependent molding of cortical columnar architecture in monkeys including macaques and marmosets (Alexander and Goldman 1978; Missler et al. 1993; Anderson et al. 1995; Shaw et al. 2008; Geschwind and Rakic 2013; Takesian and Hensch 2013), which may close around the time of peak volume. The subsequent decline in GM volume may be the result of a period of pruning and the selective elimination of synapses (Anderson et al. 1995). However, an additional contributor to a decline in the MRI signal is an increase in the fraction of “fatty” myelinated material, like axons. Consequently, the apparent shrinkage of GM may also represent the expansion of myelinated axons into the cortical neuropil (Shaw et al. 2008; Whitaker et al. 2016). Each of the 3 milestones may therefore provide insight into differential cortical organization, pruning and myelination during development.

Of the 3 milestones, the “age of peak volume” showed the smallest difference across all the cortical regions. The occipital pole was first, at approximately 5 months, and then the age increased incrementally from caudal to rostral, with the latest timing of peak volume occurring in the frontal pole and anterior temporal cortex and pole at 7.8 months. Thus, if this represents the period of experience dependent molding of cortical columnar architecture this appears to occur at a relatively similar time period across the cortex. The exceptions were in some subcortical regions including the hypothalamus, BNST, amygdala, habenula, caudate body, and raphe. Cortical regions displayed a much greater difference in the age of onset of GM decline, the age of the maximum rate of decline and the duration of that period of decline, periods likely associated with pruning and myelination. The “age of onset of the decline in volume” highlighted the late maturation (12–14 months) post puberty of the prefrontal and lateral and inferior temporal association cortices, compared with the rest of the cortex, in which onset of decline occurred at or before puberty. Of note, the association areas of the anterior cingulate cortex, including dorsal area 24 and subgenual area 25, showed a much earlier onset of decline, between 6 and 8 months, similar to primary and secondary sensory regions, suggesting that pruning and myelination processing may be engaged in these regions much earlier than neighboring prefrontal association cortex. However, the age of “maximum rate of decline,” suggestive of a period of maximal neuronal pruning/myelination, occurs very late in area 25 (19.8 m) in late adolescence, more similar in time to that of prefrontal association cortex. Indeed, this milestone is only later still in the ventral temporal lobe (20.8 m) and secondary somatosensory cortex (22 m), areas that are interconnected with area 25 (Joyce and Barbas 2018). Whilst only a small number of studies have investigated marmoset brain development in post mortem tissue and these have been limited to just a few specific cortical brain areas, there is re-assuring correspondence in the timings of periods of greatest circuitry rearrangement in those brain regions and the timing of MRI milestones described here. For example, the relatively flat MRI-derived growth trajectory of V1 after 9 months is consistent with the completion of the period of greatest circuitry rearrangement in V1 reported by (Fritschy and Garey 1986) and (Spatz et al. 1993). Also, the finding that middle temporal area and primary visual areas have similar growth trajectories and milestones is comparable to that revealed by neurofilament protein immunostaining; a marker of morphological maturation of neurons and stability of connections (Bourne and Rosa 2006). Finally, the same marker has been used to show later maturation of rostral regions of dorsolateral prefrontal cortex compared with caudal regions (Burman et al. 2007), supported by the delayed age of onset of decline in grey matter of area 46 compared with area 8 described here (compare purple and orange trajectories in Fig. 7*C*).

Additional insights into the general principles of cortical brain growth are provided by the cluster analysis of the overall developmental trajectories. Whilst we highlight the 6 cluster solution in particular, it is interesting to note that higher cluster solutions of 7–10 display a hierarchical reorganization (Fig. 8) and in all cases involve further fractionation of the prefrontal cortex involving the ventrolateral PFC (areas 45, 47) and the frontal pole (area 10) (from 6 to 7 clusters), areas 25 and 32 (from 7 to 8 clusters), area 10 (from 8 to 9 clusters), and finally areas 8 and 24 and area 13 (from 9 to 10 clusters). This indicates that not only does the prefrontal cortex, along with anterotemporal regions of association cortex, display the latest maturation but the prefrontal cortex also shows the greatest variability in developmental growth patterns. It should be noted that we do not suggest that *k* = 6 is an important biological conclusion of itself, but we have used the clustering solutions to demonstrate that our 3 chosen milestones usefully distinguish between families of trajectories chosen by a data-driven technique that does not rely on manual selection of features of interest.

### Pathophysiological Interpretation

Of the differing cortical growth patterns identified in the current study a few deserve highlighting because of their particular novelty and relevance to symptoms of neuropsychiatric disorders and susceptibility to stress during development. The rapid onset of decline in the cingulate cortex occurring at approximately the same time as that of the occipital lobe suggests that maturational processes start early throughout this region. However, unlike the occipital and dorsal anterior and posterior cingulate cortices, the maturational processes in the ventral anterior cingulate cortex, primarily area 25, extend over the entire pubertal and postpubertal periods. This may indicate that the subgenual cingulate cortex is particularly vulnerable to external stressors for extended periods of development and may explain its prominent role in models of depression (Drevets et al. 2008; Johansen-Berg et al. 2008). Other areas showing an overall similar growth trajectory to subgenual area 25 include the more anterior ventromedial areas 14 and 32, medial orbital regions 11 and 13, areas 9 and 46 and secondary somatosensory association cortex, regions with which area 25 is directly connected (Joyce and Barbas 2018). In addition, this trajectory pattern best described those of the ventromedial caudate, accumbens, basolateral amygdala, mediodorsal thalamus, and medial raphe area, all regions with extensive connectivity with medial orbital and ventromedial regions of PFC and involved in the regulation of emotion (Price and Drevets 2012; Joyce and Barbas 2018).

The other cluster within *k* = 6 clusters to involve prefrontal regions included frontal polar area 10, and ventrolateral PFC regions 45 and 47, alongside the full extent of the insula cortex and neighboring proisocortex in the temporal pole. The inclusion of the auditory parabelt region is consistent with the known connections of this region with vocalization processing within ventrolateral PFC regions in macaques (Plakke and Romanski 2014). Moreover, vocalizations induced by periaqueductal grey stimulation activate the vlPFC region in other new world monkeys, for example, tamarinds (Jurgens et al. 1996). These findings concur with those of (Raznahan et al. 2011), which, by studying the default mode and task positive networks in humans, provided evidence for close associations between functional and maturational coupling.

Together the findings presented here, in particular the heterogeneity of developmental trajectories within the prefrontal and cingulate cortices, provides important new insight into the distinct stages of development at which these different prefrontal/cingulate circuits, and hence different cognitive and emotional functions, may be at their most vulnerable to stress-related psychological, physiological and immunological impact. This will guide future developmental stress models of psychiatric disorders, highlighting different windows of sensitivity for targeting stressful interventions. Such models have the potential to reveal distinct behavioral phenotypes arising from these different developmental epochs that may cause a specific spectrum of neuropsychiatric symptoms. In addition, knowledge of the timing of milestones in distinct brain regions will guide not only future post mortem studies but also further imaging studies targeting these relevant developmental epochs to characterize the underlying cellular processes that these milestones represent.

### Considerations of Growth Curve Modeling Across Studies

In recently published marmoset studies (Seki et al. 2017; Uematsu et al. 2017) the MRI is at a lower spatial resolution than the present study and importantly a biexponential model (i.e., $V=Ae^{\alpha t}-Be^{\beta t}$, for the volume V of a brain region at age t) is fitted to the data after removing subject effects, in contrast to the cubic spline approach used here. The biexponential growth model can only support a single turning point (at age $t=\frac{1}{\beta -\alpha }\;\ln\left(\alpha A/\beta B\right)$) with all brain structures showing rapid initial postnatal volume growth with relative stability in adulthood. The constraint of a single turning point restricts flexibility in the crucial stages between infancy and adolescence (which are also where many neuropsychiatric problems become apparent). Thus, this model is unable to capture the additional features that we have shown clearly differentiate growth patterns of primary and association areas of cortex. Other studies in the marmoset have a more limited scope, for example, focussing exclusively on corpus callosum measurements (Sakai et al. 2017) or gross cortical divisions (Seki et al. 2017).

Similarly, the low-order polynomial models typically adopted in human studies (Shaw et al. 2008), such as linear, quadratic, and cubic curves, which allow 0, 1, or 2 turning points respectively, also do not allow as much flexibility to match features in growth curves as afforded by cubic splines. In addition, the parameters from such models can be highly variable depending on seemingly irrelevant factors such as the range in ages of the included subjects (Fjell et al. 2010) and by their nature have inappropriate limiting behavior for very young and very old subjects. Although polynomial models may serve as suitable interpolants over limited age ranges, they are incapable of accurately modeling more complicated features such as the rapid initial growth followed by a delayed onset of decline prior to the relative stability seen in adulthood that our data show.

### Study Limitations

Two processes are responsible for GM volume reductions reported here. Proton density in myelinated tissue is approximately 10% lower and bound protons in myelin layers have a shortened T2, leading to lower signal intensity in the present MRI protocol (Tofts 2003). As the volume fraction of myelin increases, the signal becomes hypointense and such tissues are given a greater WM weighting and a lesser GM weighting in the segmentation process.

Other processes can cause signal reductions that could increase the likelihood that voxels are labeled as WM, such as iron deposition or reduction in water content. A more sophisticated imaging protocol, for example multiparametric mapping (Helms et al. 2008; Weiskopf et al. 2013) or the incorporation of diffusion-tensor imaging measurements could more reliably capture WM. Nevertheless, the WM maps produced are consistent with the known anatomy and the complementary techniques of visual inspection, TBM, and ROI-based trajectory analysis give consistent results.

## Conclusions

We present the first detailed survey of regional growth trajectories in a monkey brain from infancy to adulthood. Three milestones of development are identified that together describe the overall growth trajectories and highlight distinct clusters of cortical brain regions with markedly different trajectories. Not only does the prefrontal cortex show late adolescent maturational changes consistent with those already reported in humans and nonhuman primates but we show for the first time that the prefrontal cortex also has the greatest intraregional variability of growth patterns of relevance to the varied symptomatology of neuropsychiatric disorders which have their onset during development. In addition, consideration of the particular groupings of brain regions within clusters, reveal evidence for functional and maturational coupling.

## Supplementary Material

Supplementary Data
