# Supplementary material for: Trajectories and Milestones of Cortical and Subcortical Development of the Marmoset Brain From Infancy to Adulthood
Source: Cereb Cortex. 2018 Oct 11;28(12):4440–53. doi: 10.1093/cercor/bhy256 (PMC6215464; doi:10.1093/cercor/bhy256)
Supplement: Supplementary Data [file bhy256_suppl.zip › bhy256_Supplementary.pdf]

Trajectories and milestones of cortical and subcortical development of the marmoset brain from infancy to adulthood

S. J. Sawiak[1,2*], Y. Shiba[1,3], L. Oikonomidis[1,3], C. P. Windle[3], A. M. Santangelo[3], H. Grydeland[4,5], G. Cockcroft[1,3], E. T. Bullmore†[1,2,4,6], A. C. Roberts†[1,3]

[1]Behavioural and Clinical Neuroscience Institute, University of Cambridge, Downing Site, CB2 3EB
[2]Wolfson Brain Imaging Centre, University of Cambridge, Box 65 Addenbrooke's Hospital, Cambridge CB2 0QQ
[3]Department of Physiology, Development and Neuroscience, University of Cambridge, Downing Street, Cambridge, CB2 3EB
[4]Department of Psychiatry, University of Cambridge, Cambridge CB2 0SZ
[5] Research Group for Lifespan Changes in Brain and Cognition, Department of Psychology, University of Oslo, 0317 Oslo, Norway
[6]ImmunoPsychiatry, GlaxoSmithKline Research and Development, Stevenage, SG1 2NY

Supplemental Material
Table of Contents

1. **Supplemental Figures**

   Fig S1 Voxel-based assessment of asymmetry at 3, 12 and 21 months.
   Fig S2 Significant differences of milestones between clusters for clustering solutions k=2…10.

2. **Supplemental Table 1**
   Complete listing of milestones for each brain region

3. **Abbreviations for brain regions**

4. **Growth trajectories and milestones**

5. **Supplementary Movie 1**

Supplementary Figure 1

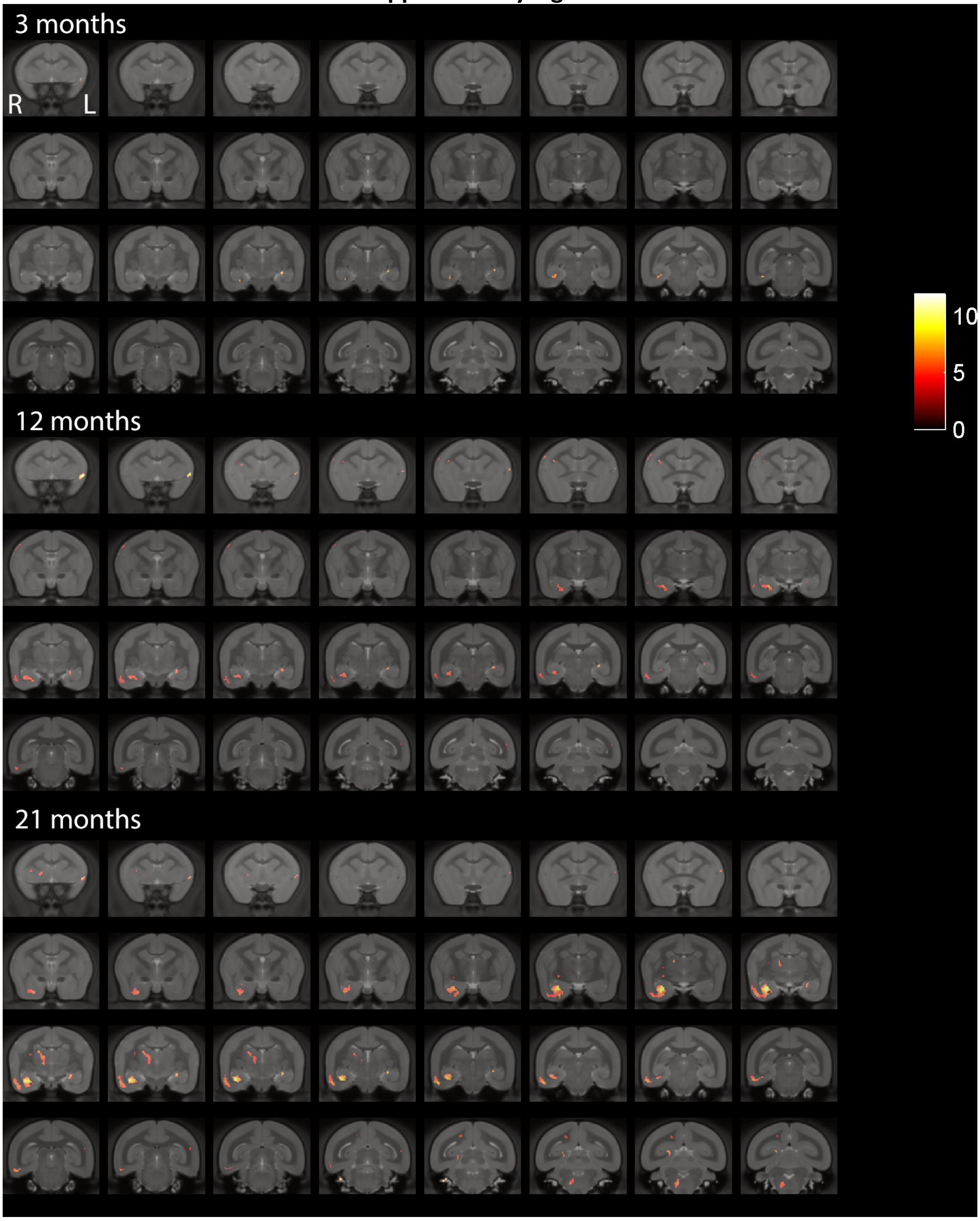

Supplementary Figure 1 Voxel-based maps of asymmetry indicating regions significantly larger than the contralateral side at 3, 12 and 21 months. Colour bar indicates Student's t-score. All results are significant at ($p < 0.05$, false-discovery rate corrected).

Supplementary figure 2

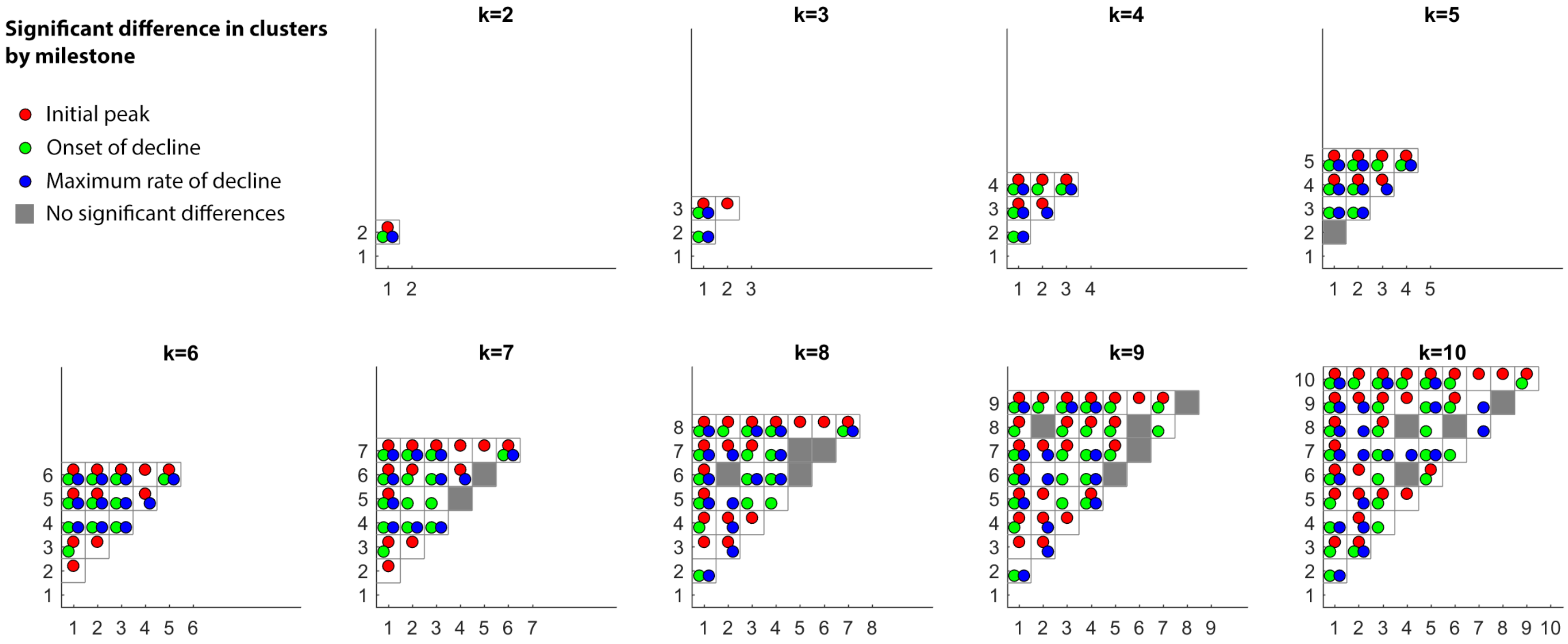

Supplementary Figure 2

Comparisons between milestones (age at peak volume, at the onset of decline and at the maximum rate of decline) for each cluster for k-means clustering solutions (k=2-10). k=6 is the largest solution where each cluster is significantly different from each other in at least one milestone. Significance was determined using a two-tailed Student's *t*-test ($p < 0.05$).

Supplementary Table 1

The age at which the milestones are reached for each structure. The second milestone is the upper limit of the 95% confidence interval for the peak.

Structure	Abbrev.	Peak volume I (months)	95% CI II (months)	Maximum rate of decline III (months)
Areas 1 and 2	A1-2	5.76	[5.15, 6.55]	16.05
Area 3	A3	5.86	[5.28, 6.78]	16.02
Area 4	A4	6.03	[5.50, 7.09]	15.90
Area 6	A6	6.01	[5.43, 7.42]	15.82
Area 8	A8	5.83	[5.11, 12.77]	15.74
Area 9	A9	6.54	[3.18, 12.73]	16.57
Area 10	A10	6.88	[5.54, 14.39]	15.86
Area 11	A11	6.19	[4.29, 13.96]	17.49
Area 13	A13	6.10	[5.28, 11.72]	13.92
Area 14	A14	5.37	[3.74, 6.47]	17.05
Area 19	A19	5.78	[5.37, 6.19]	8.21
Area 23	A23	5.55	[4.88, 6.05]	8.01
Area 24	A24	5.75	[5.06, 6.48]	15.24
Area 25	A25	5.33	[4.02, 6.03]	19.81
Area 29	A29	5.65	[5.08, 6.10]	14.57
Area 30	A30	5.65	[5.10, 6.07]	14.72
Area 31	A31	5.23	[3.93, 5.84]	7.90
Area 32	A32	5.38	[3.53, 12.65]	19.37
Area 35	A35	7.50	[6.36, 13.97]	16.31
Area 36	A36	7.34	[6.29, 14.23]	18.70
Area 45	A45	6.01	[5.17, 12.02]	15.06
Area 46	A46	5.91	[4.39, 13.09]	17.56
Area 47	A47	5.96	[5.19, 11.90]	15.17
Agranular insular cortex	AI	6.44	[5.74, 12.69]	15.85
Dysgranular insular cortex	DI	6.25	[5.62, 12.70]	15.68
Granular insular cortex	GI	6.13	[5.53, 12.26]	16.00
Ant. intraparietal area	IP	5.87	[5.41, 6.38]	8.22
Amygdalopiriform transition area	APir	7.79	[6.65, 13.83]	16.07
Auditory cortex primary area	AuA1	6.42	[5.71, 8.68]	13.81
Auditory cortex belt	AuAL	6.46	[5.80, 8.31]	14.53
Auditory cortex parabelt	AuCPB	6.43	[5.77, 12.58]	14.58
Entorhinal cortex	Ent	7.28	[6.34, 14.27]	16.80
Fundus of sup. temporal sulcus	FST	6.68	[5.88, 12.86]	14.28
Proisocortex	TPro/IPro	6.63	[5.83, 12.99]	15.78
Med. sup. temporal area	MST	5.91	[5.43, 6.53]	8.24
Orbital proisocortex	OPAl	5.88	[5.17, 12.20]	14.52
Occipito-parietal trans area	OPt	5.95	[5.63, 6.35]	8.16
Parietal area PE	PE	5.56	[4.79, 6.08]	7.96

Structure	Abbrev.	Peak volume I (months)	95% CI II (months)	Maximum rate of decline III (months)
Parietal area PF	PF	5.78	[5.39, 6.28]	16.15
Parietal area PG	PG	5.86	[5.52, 6.25]	8.15
Parietal area PG medial part	PGM	5.74	[5.22, 6.22]	8.18
Piriform	Pir	7.49	[6.32, 13.76]	17.22
Proisocortical motor region	ProM	5.80	[4.95, 12.56]	15.43
Secondary somatosensory	S2E	5.85	[4.68, 11.71]	21.94
Temporal area TE1/2	TE1	7.04	[6.17, 12.35]	14.79
Temporal area TE3	TE3	7.42	[6.30, 12.57]	14.74
Ventral temporal lobe	TF	7.81	[6.66, 13.64]	20.76
Visual area 1	V1	4.95	[2.51, 5.73]	8.15
Visual area 2	V2	5.70	[5.12, 6.19]	8.34
Visual area 3	V3	6.19	[5.78, 6.69]	8.57
Visual area 4	V4	6.32	[5.84, 6.91]	14.03
Visual area 5	V5	5.99	[5.51, 6.58]	8.53
Visual area 6	V6	5.38	[4.65, 5.92]	7.79
Subcortical/allocortical areas				
Caudate body	CaudBody	11.78	[6.04, 13.42]	17.29
Dorsolateral caudate	DLCaud	6.24	[5.74, 7.19]	15.97
Ventromedial caudate	VMCaud	6.39	[5.91, 7.76]	16.17
Putamen	Put	6.74	[6.05, 12.76]	14.74
Accumbens	Acb	5.78	[5.34, 6.46]	15.79
Central amygdala	AmygCe	13.00	[12.02, 14.10]	22.86
Basolateral amygdala	AmygBL	12.44	[5.92, 13.51]	15.9
Cerebellum	Cereb	9.93	[7.71, 16.76]	21.58
Ant. Hippocampus	antHIPP	13.06	[12.02, 14.05]	15.73
Dorsal raphe	d raphe	13.36	[7.22, 20.70]	22.07
Medial raphe	MRaphe	13.03	[7.20, 14.49]	16.06
BNST	BNST	15.47	[8.08, 23.98]	20.65
Ant. hypothalamus	antHypo	13.94	[12.70, 14.98]	16.32
Mediodorsal thalamus	MD Thal	6.85	[6.10, 8.04]	16.63
Habenula	Hab	13.51	[7.48, 15.76]	17.43
Lateral septum	LSeptum	6.30	[5.74, 7.65]	16.49

Abbreviations used

Cortical structures

A1-2 areas 1 and 2 of cortex; A10 area 10 of cortex; A11 area 11 of cortex; A13 area 13 of cortex (including, lateral, medial and 13a and b); A14 (area 14 of cortex including caudal and rostral part); A19 area 19 of cortex (dorsointermediate and medial parts); A23 area 23 (ventral part; A23a, b and c); A24 area 24 of cortex (including 24a, b, c, d); A25 area 25 of cortex; A29 area 29 of cortex (including areas 29a-d), A30 area 30 of cortex; A31 area 31 of cortex; A32 area 32 of cortex (including ventral part); A35 area 35 of cortex; A36 area 36 of cortex; A3 area 3 of cortex (somatosensory, (including 3a and 3b); A45 area 45 of cortex; A46 area 46 of cortex (including dorsal and ventral parts); A47 area 47 (old 12) of cortex (including lateral, medial and orbital parts); A4ab area 4 of cortex (primary motor; including parts a and b and c); A6 area 6 of cortex (including dorsocaudal, medial, dorsorostral and ventral parts a and b); A8 area 8 of cortex (including caudal, dorsal and ventral aspects, parts a and b); A9 area 9 of cortex; AI agranular insular cortex; AIP anterior intraparietal area of cortex; APir amygdalopiriform transition area; AuA1 auditory cortex primary area (including rostral and rostrotemporal areas); auditory cortex belt: (AuAL auditory cortex anterolateral area; AuCL auditory cortex caudolateral area; AuCM auditory cortex caudomedial area; AuML auditory cortex middle lateral area; AuRM auditory cortex rostromedial area) auditory cortex parabelt: (AuCPB auditory cortex caudal parabelt area; AuRPB auditory cortex rostral parabelt; AuRTM auditory cortex rostrotemporal medial area, STR superior temporal rostral area (cortex)); DI dysgranular insular cortex; Ent entorhinal cortex; FST fundus of superior temporal sulcus area of cortex; GI granular insular cortex; Pro proisocortex; LIP lateral intraparietal area of cortex; MIP medial intraparietal area of cortex; MST medial superior temporal area of cortex; OPAl orbital periallocortex; OPro orbital proisocortex; OPt occipito-parietal transitional area of cortex; PE parietal area PE (including caudal part); PF parietal area PF (cortex); PG parietal area PG; PGM parietal area PG medial part (cortex); Pir piriform cortex; ProM proisocortical motor region (precentral opercular cortex); ProSt prostriate area; S2 secondary somatosensory cortex (including external and internal parts, rostral and parietal ventral areas). TE temporal areas TE1 and 2 (inferior temporal cortex, including occipital part); TE3 temporal area TE3 (inferior temporal cortex); ventral temporal lobe (including areas TF and its occipital part, temporal areas TH and TL); V1 primary visual cortex; V2 visual area 2; V3 visual area 3 (ventrolateral posterior area including area V3a); V4 visual area 4 (ventrolateral anterior area, including middle temporal crescent); V5 visual area 5 (middle temporal area); V6 visual area 6 (dorsomedial area and V6a).

Sub/allocortical structures

DLCaud, dorsolateral caudate nucleus; Put, putamen; VMCaud, ventromedial caudate; Acb, nucleus accumbens; BL Amyg, basolateral amygdala; antHIPP, anterior hippocampus; DRaphe, dorsal raphe nucleus; MRaphe, medial raphe nucleus; Caud, caudate body; BNST, bed nucleus of the stria terminalis; antHypo, anterior hypothalamus; MD Thal, medial dorsal thalamus; Hab, habenula; LSeptum, lateral septum; AmygCe, central nucleus of the amygdala.

Growth trajectories and milestones

There follows, for each cortical and subcortical brain region studied, plots of the trajectory with its first derivative, indicating the milestones. A schematic of the region in the brain is shown with its abbreviation. The timing of the peak is given followed by its 95% confidence interval and the maximum rate of decline.

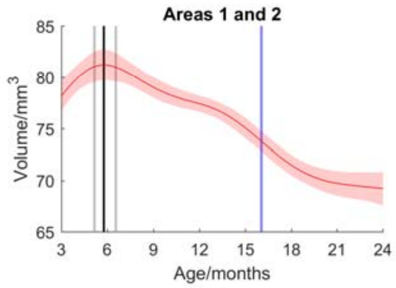

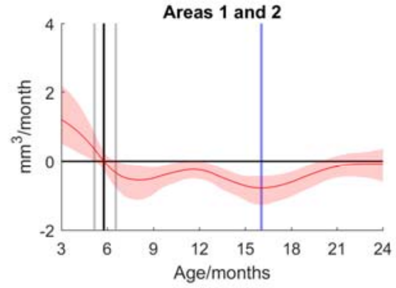

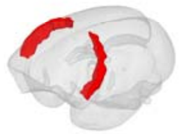

Areas 1 and 2
A1-2

Peak at 5.76 months [5.15, 6.55]
Maximum rate at 16.05 months.

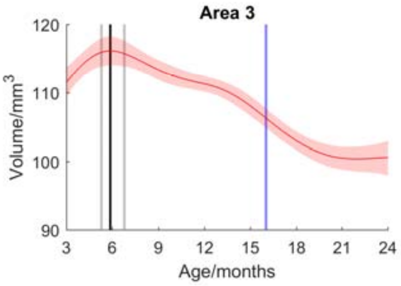

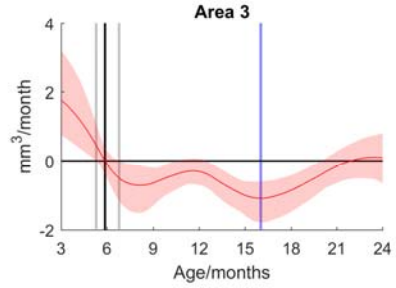

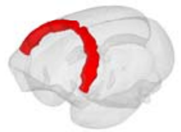

Area 3
A3

Peak at 5.86 months [5.28, 6.78]
Maximum rate at 16.02 months.

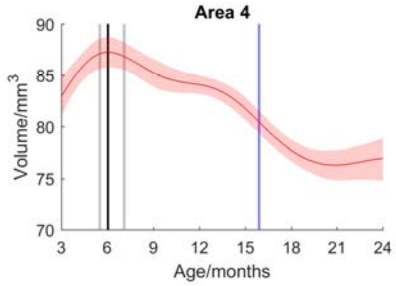

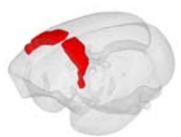

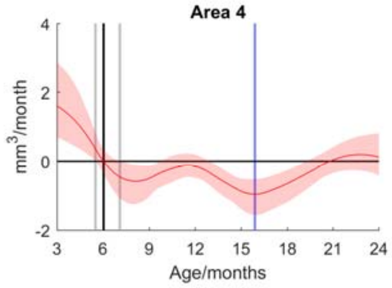

Area 4
A4

Peak at 6.03 months [5.50, 7.09]
Maximum rate at 15.90 months.

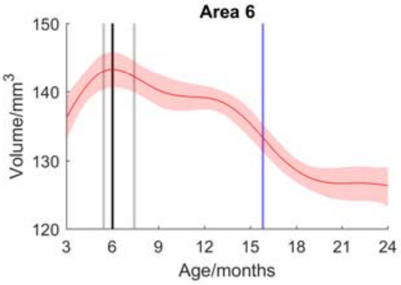

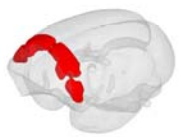

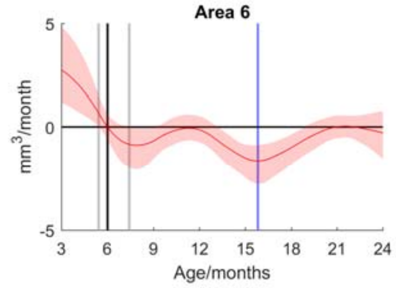

Area 6
A6

Peak at 6.01 months [5.43, 7.42]
Maximum rate at 15.82 months.

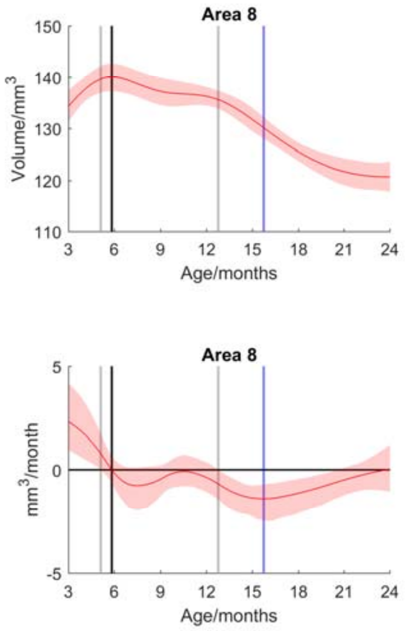

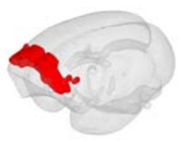

Area 8
A8

Peak at 5.83 months [5.11, 12.77]
Maximum rate at 15.74 months.

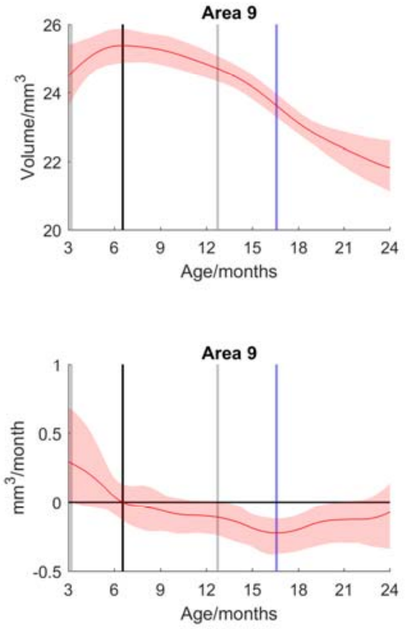

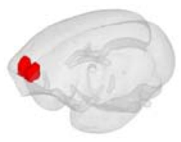

Area 9
A9

Peak at 6.54 months [3.18, 12.73]
Maximum rate at 16.57 months.

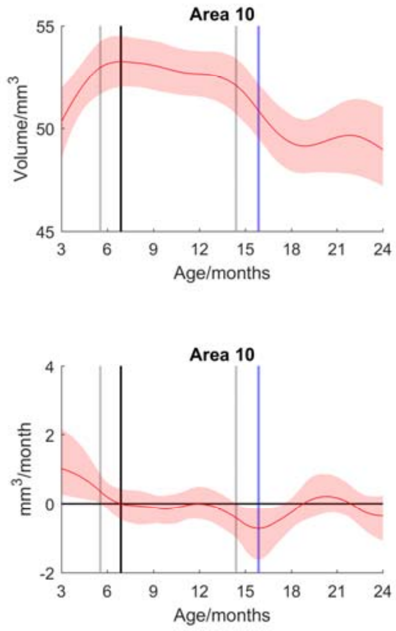

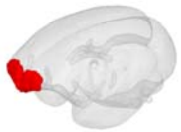

Area 10
A10

Peak at 6.88 months [5.54, 14.39]
Maximum rate at 15.86 months.

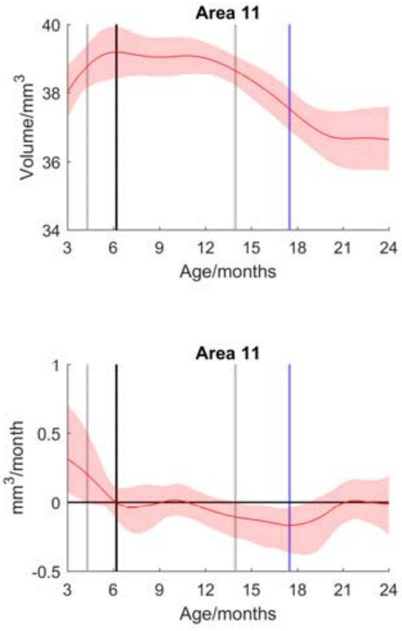

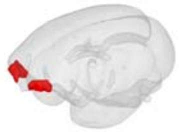

Area 11
A11

Peak at 6.19 months [4.29, 13.96]
Maximum rate at 17.49 months.

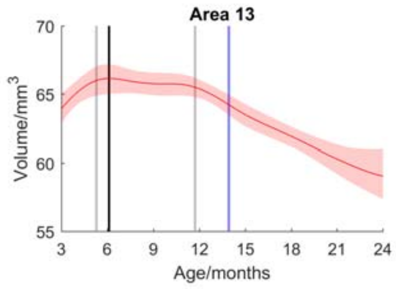

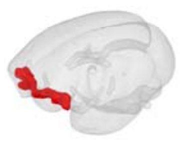

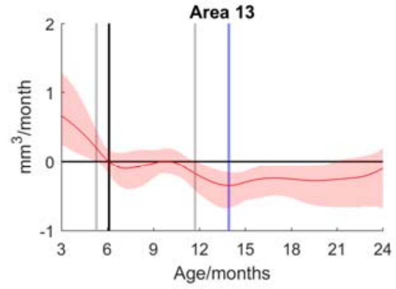

Area 13
A13

Peak at 6.10 months [5.28, 11.72]
Maximum rate at 13.92 months.

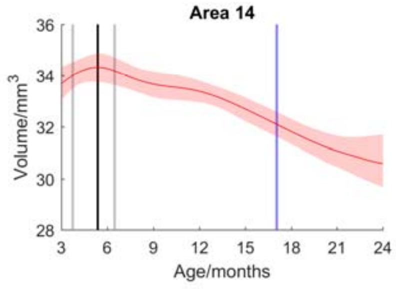

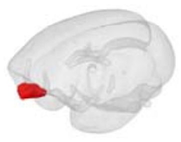

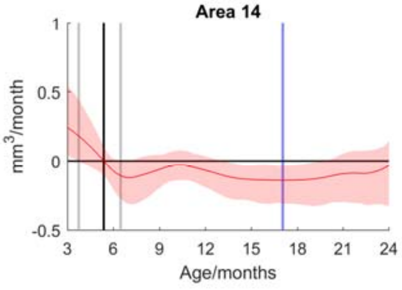

Area 14
A14

Peak at 5.37 months [3.74, 6.47]
Maximum rate at 17.05 months.

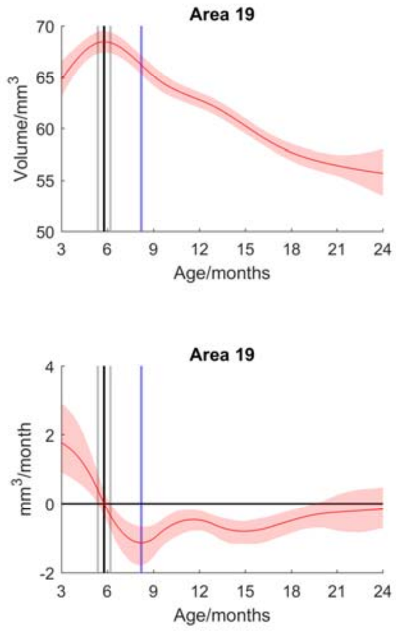

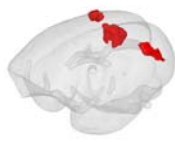

Area 19
A19

Peak at 5.78 months [5.37, 6.19]
Maximum rate at 8.21 months.

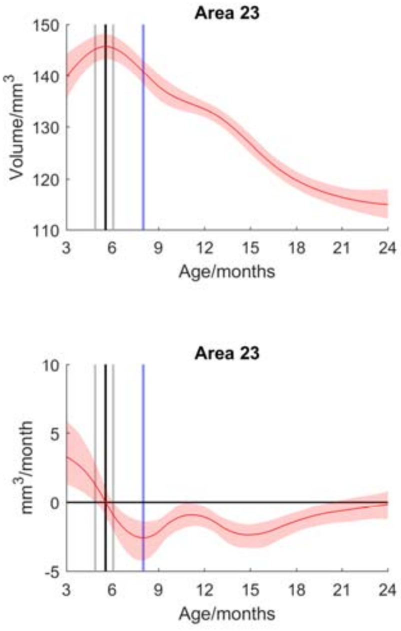

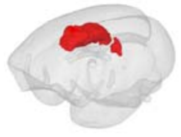

Area 23
A23

Peak at 5.55 months [4.88, 6.05]
Maximum rate at 8.01 months.

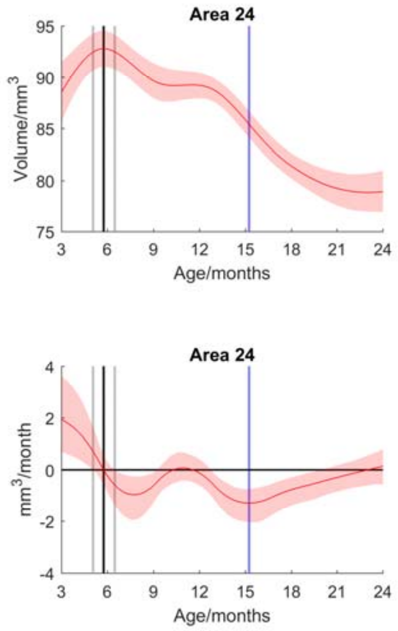

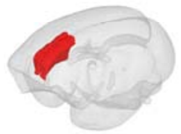

Area 24
A24

Peak at 5.75 months [5.06, 6.48]
Maximum rate at 15.24 months.

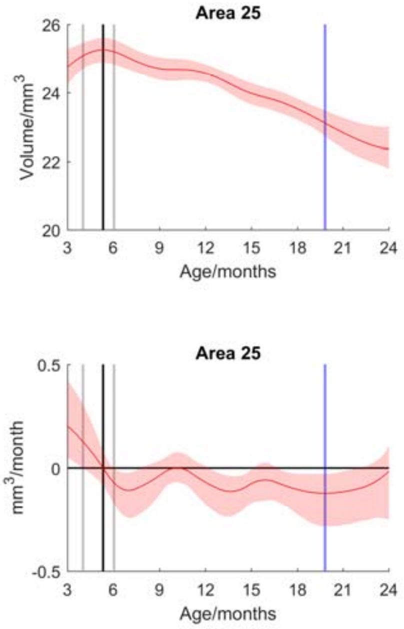

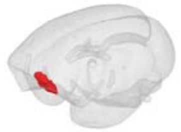

Area 25
A25

Peak at 5.33 months [4.02, 6.03]
Maximum rate at 19.81 months.

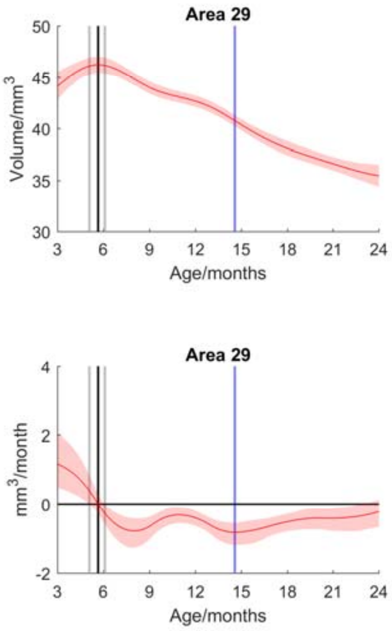

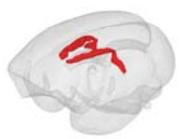

Area 29
A29

Peak at 5.65 months [5.08, 6.10]
Maximum rate at 14.57 months.

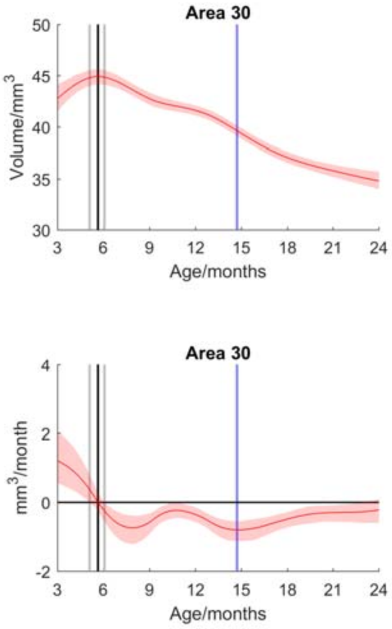

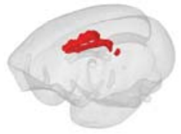

Area 30
A30

Peak at 5.65 months [5.10, 6.07]
Maximum rate at 14.72 months.

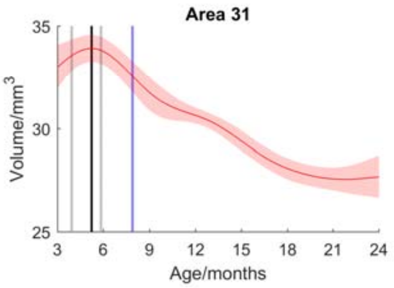

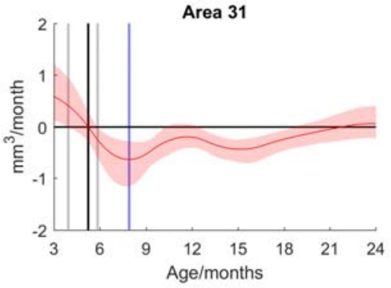

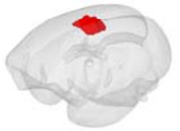

Area 31
A31

Peak at 5.23 months [3.93, 5.84]
Maximum rate at 7.90 months.

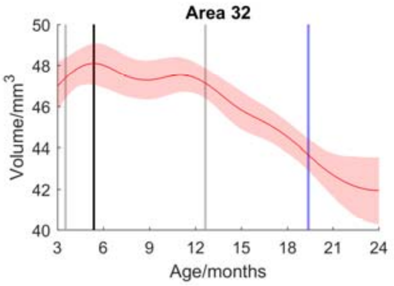

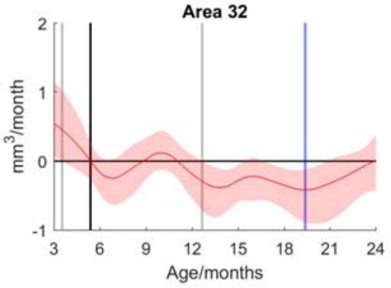

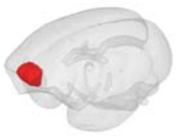

Area 32
A32

Peak at 5.38 months [3.53, 12.65]
Maximum rate at 19.37 months.

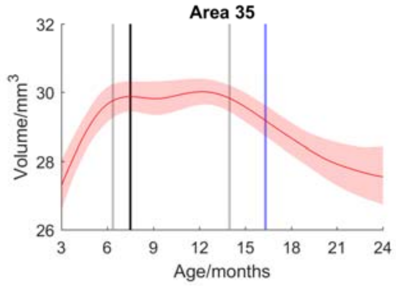

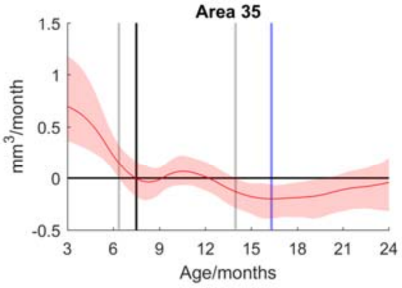

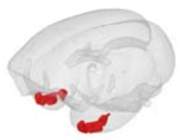

Area 35
A35

Peak at 7.50 months [6.36, 13.97]
Maximum rate at 16.31 months.

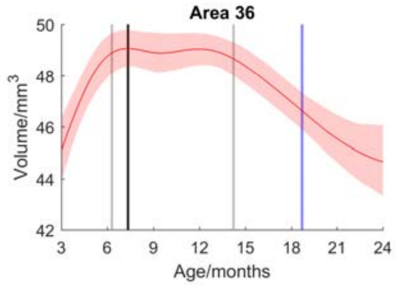

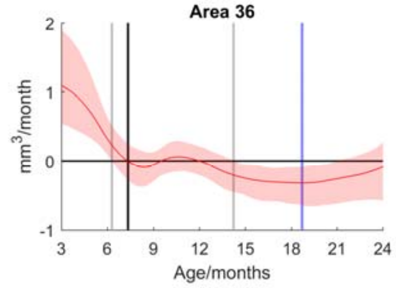

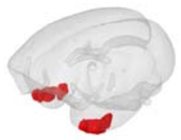

Area 36
A36

Peak at 7.34 months [6.29, 14.23]
Maximum rate at 18.70 months.

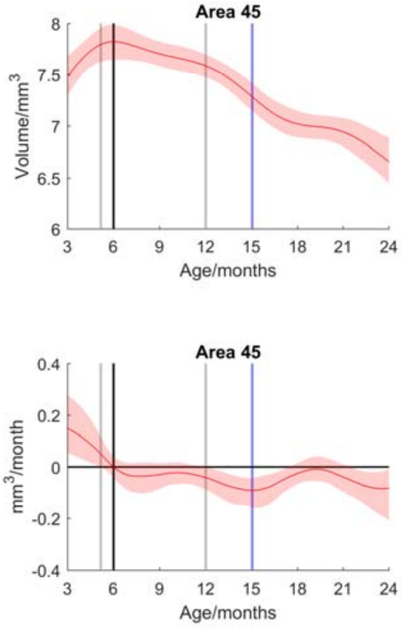

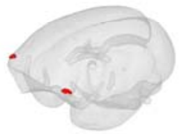

Area 45
A45

Peak at 6.01 months [5.17, 12.02]
Maximum rate at 15.06 months.

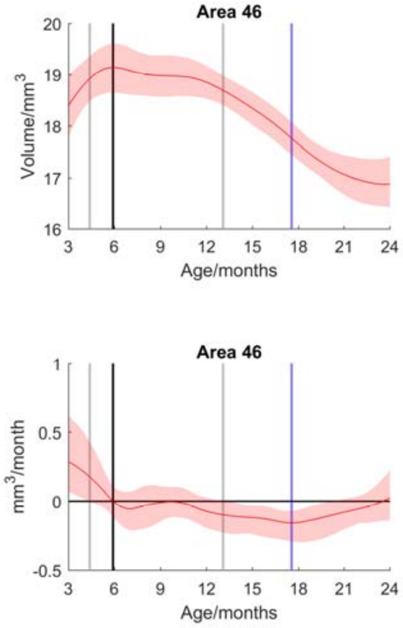

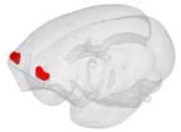

Area 46
A46

Peak at 5.91 months [4.39, 13.09]
Maximum rate at 17.56 months.

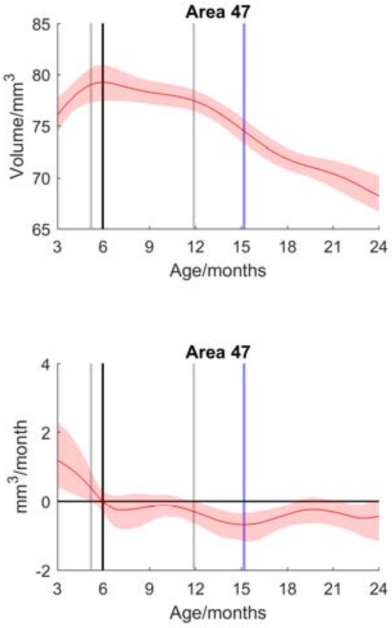

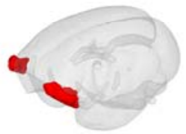

Area 47
A47

Peak at 5.96 months [5.19, 11.90]
Maximum rate at 15.17 months.

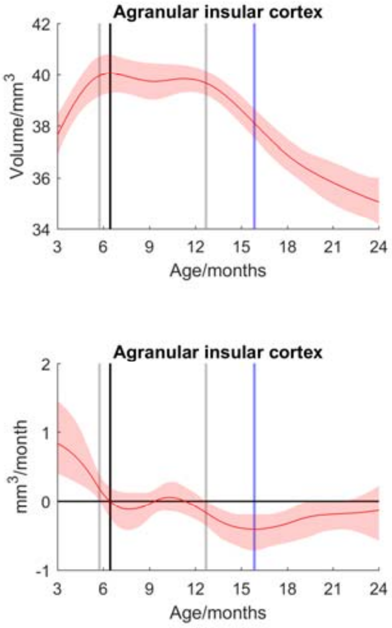

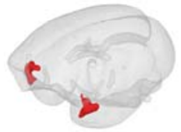

Agranular insular cortex
AI

Peak at 6.44 months [5.74, 12.69]
Maximum rate at 15.85 months.

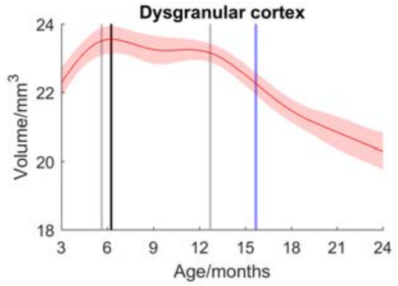

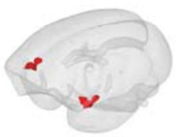

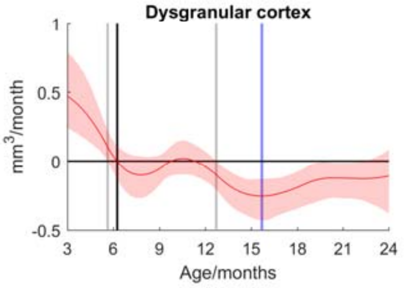

Dysgranular cortex
DI

Peak at 6.25 months [5.62, 12.70]
Maximum rate at 15.68 months.

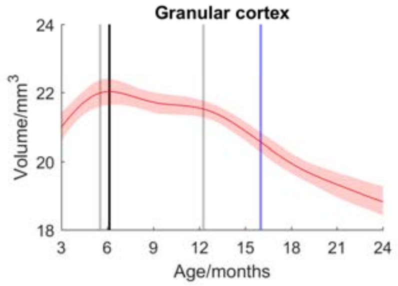

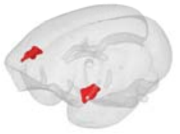

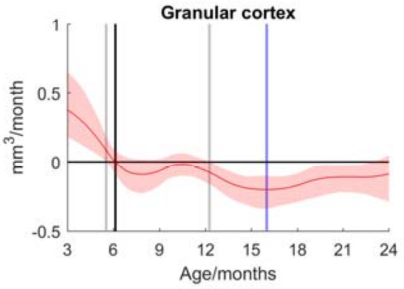

Granular cortex
GI

Peak at 6.13 months [5.53, 12.26]
Maximum rate at 16.00 months.

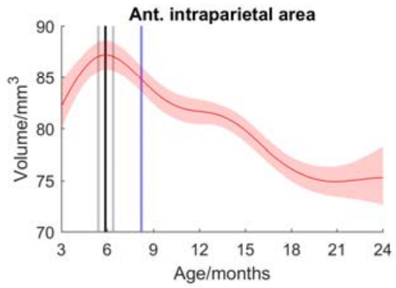

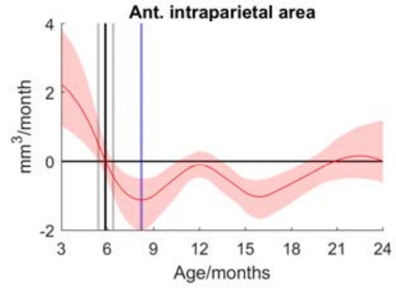

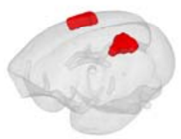

Ant. intraparietal area
IP

Peak at 5.87 months [5.41, 6.38]
Maximum rate at 8.22 months.

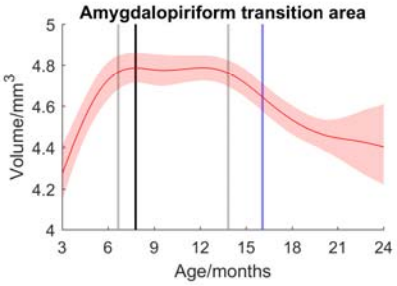

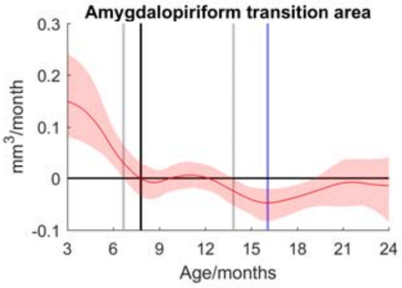

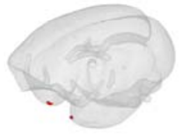

Amygdalopiriform transition area
APir

Peak at 7.79 months [6.65, 13.83]
Maximum rate at 16.07 months.

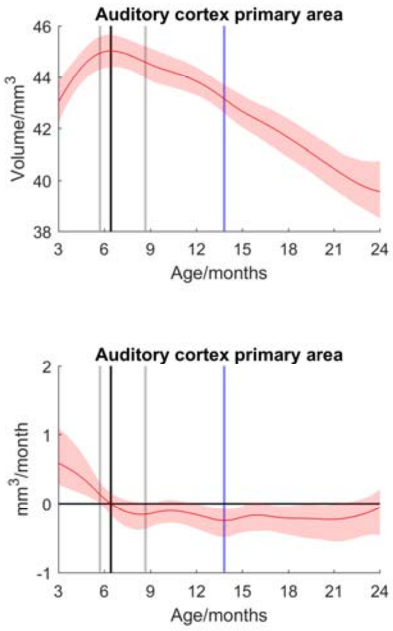

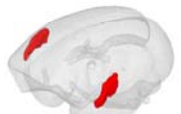

Auditory cortex primary area
AuA1

Peak at 6.42 months [5.71, 8.68]
Maximum rate at 13.81 months.

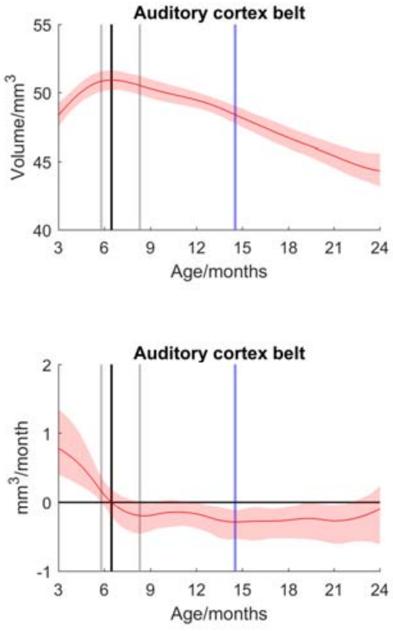

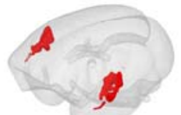

Auditory cortex belt
AuAL

Peak at 6.46 months [5.80, 8.31]
Maximum rate at 14.53 months.

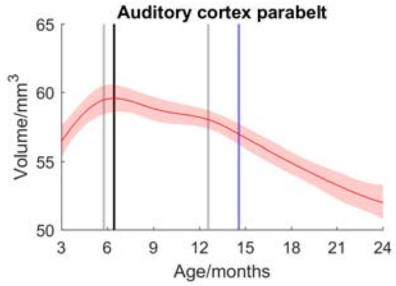

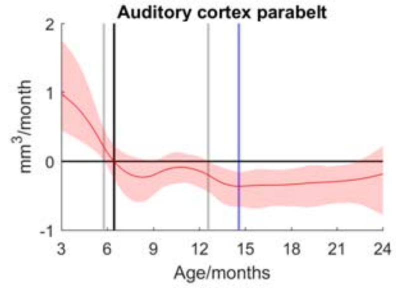

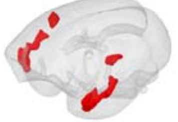

Auditory cortex parabelt
AuCPB

Peak at 6.43 months [5.77, 12.58]
Maximum rate at 14.58 months.

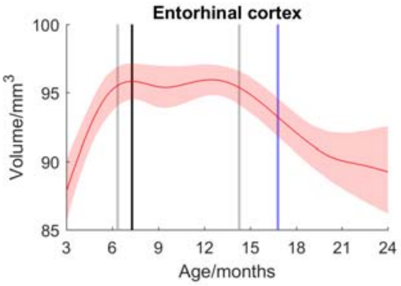

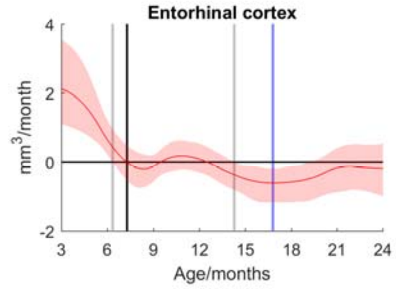

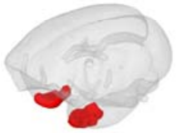

Entorhinal cortex
Ent

Peak at 7.28 months [6.34, 14.27]
Maximum rate at 16.80 months.

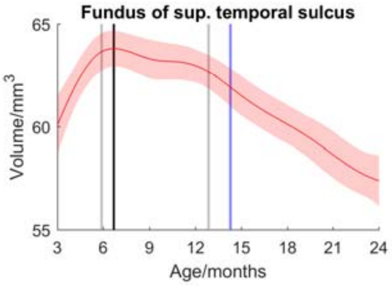

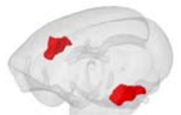

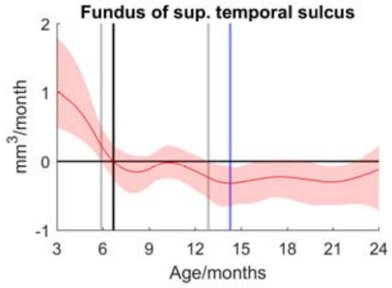

Fundus of sup. temporal sulcus
FST

Peak at 6.68 months [5.88, 12.86]
Maximum rate at 14.28 months.

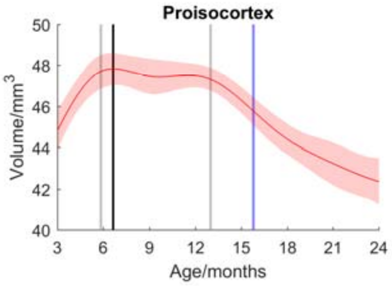

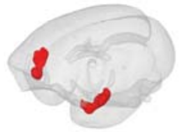

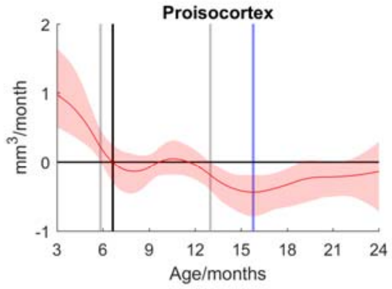

Proisocortex
TPro/IPro

Peak at 6.63 months [5.83, 12.99]
Maximum rate at 15.78 months.

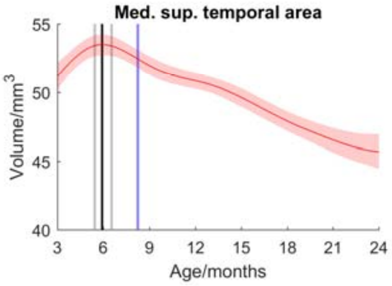

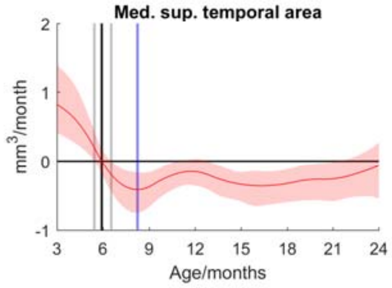

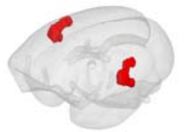

Med. sup. temporal area
MST

Peak at 5.91 months [5.43, 6.53]
Maximum rate at 8.24 months.

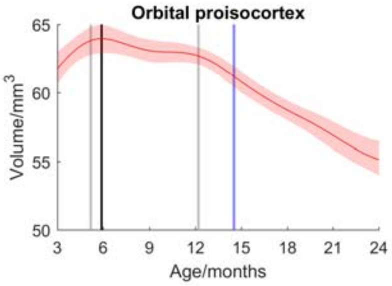

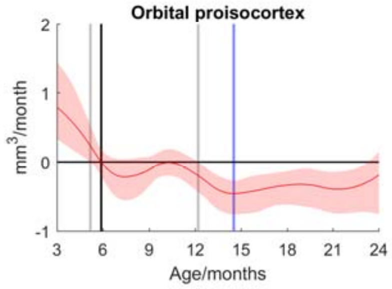

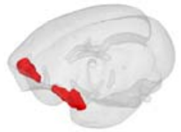

Orbital proisocortex
OPAI

Peak at 5.88 months [5.17, 12.20]
Maximum rate at 14.52 months.

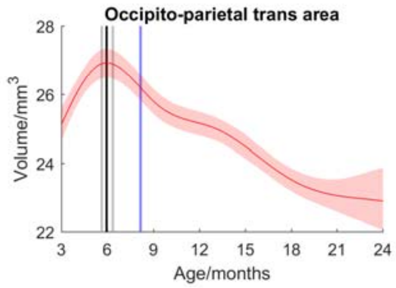

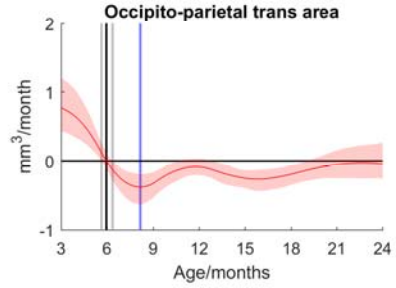

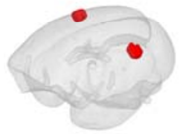

Occipito-parietal trans area
OPt

Peak at 5.95 months [5.63, 6.35]
Maximum rate at 8.16 months.

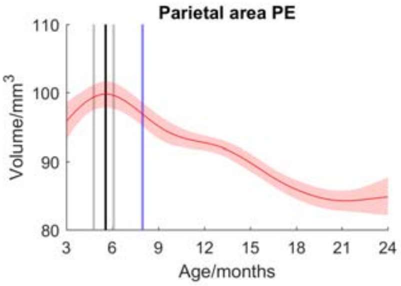

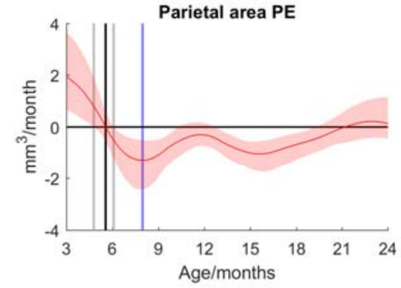

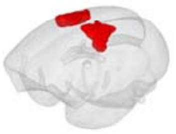

Parietal area PE
PE

Peak at 5.56 months [4.79, 6.08]
Maximum rate at 7.96 months.

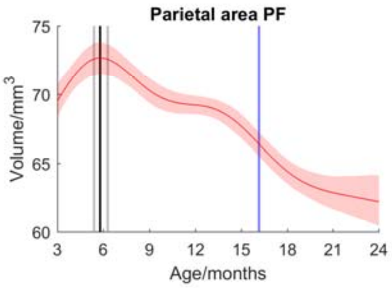

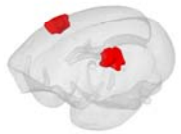

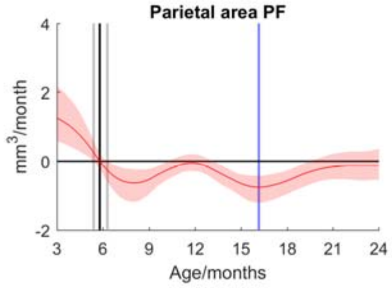

Parietal area PF
PF

Peak at 5.78 months [5.39, 6.28]
Maximum rate at 16.15 months.

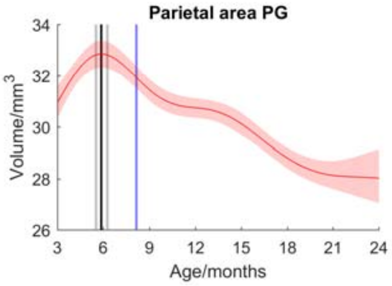

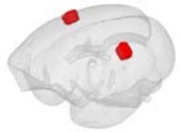

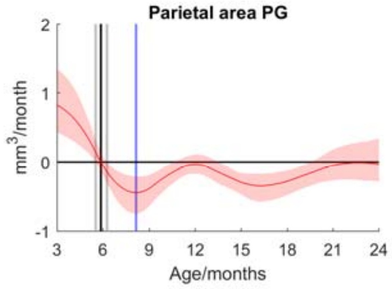

Parietal area PG
PG

Peak at 5.86 months [5.52, 6.25]
Maximum rate at 8.15 months.

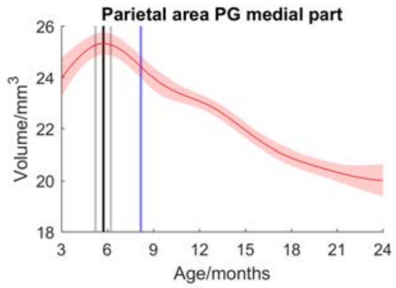

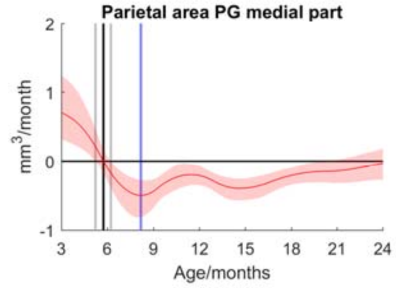

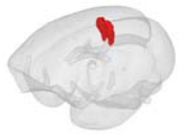

Parietal area PG medial part
PGM

Peak at 5.74 months [5.22, 6.22]
Maximum rate at 8.18 months.

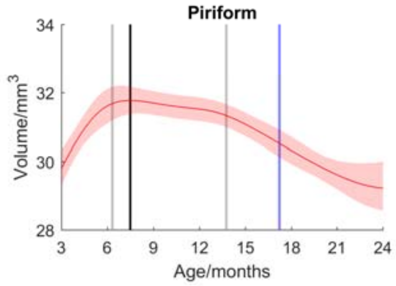

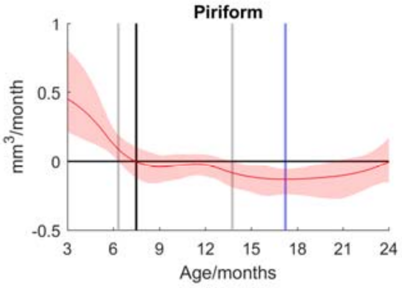

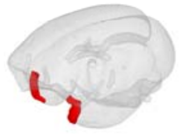

Piriform
Pir

Peak at 7.49 months [6.32, 13.76]
Maximum rate at 17.22 months.

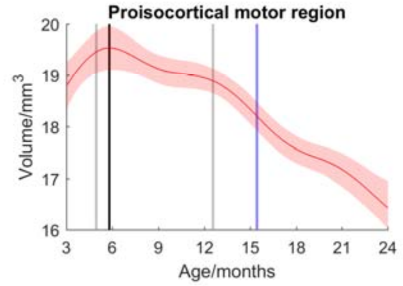

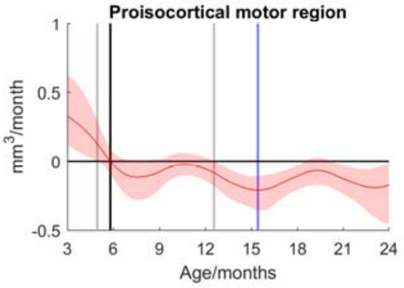

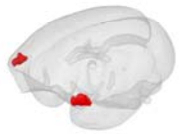

Proisocortical motor region
ProM

Peak at 5.80 months [4.95, 12.56]
Maximum rate at 15.43 months.

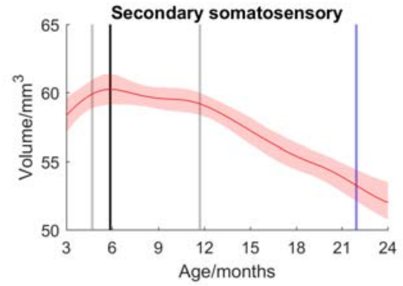

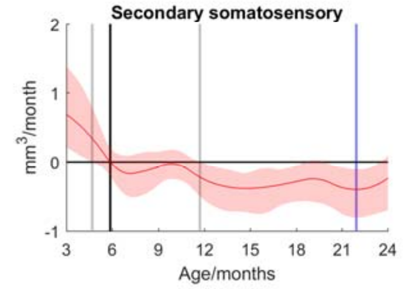

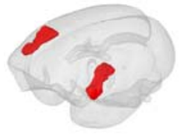

Secondary somatosensory
S2E

Peak at 5.85 months [4.68, 11.71]
Maximum rate at 21.94 months.

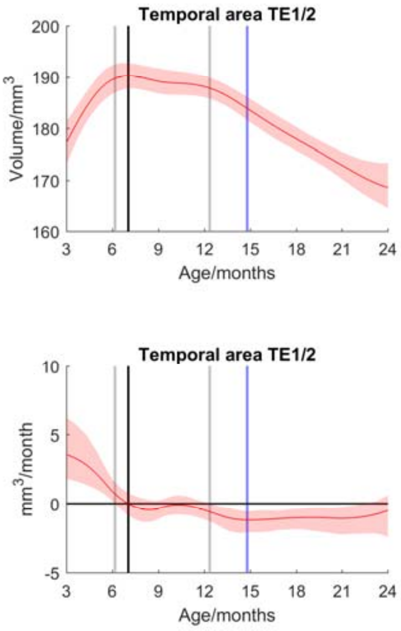

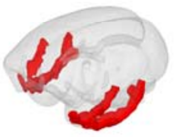

Temporal area TE1/2
TE1

Peak at 7.04 months [6.17, 12.35]
Maximum rate at 14.79 months.

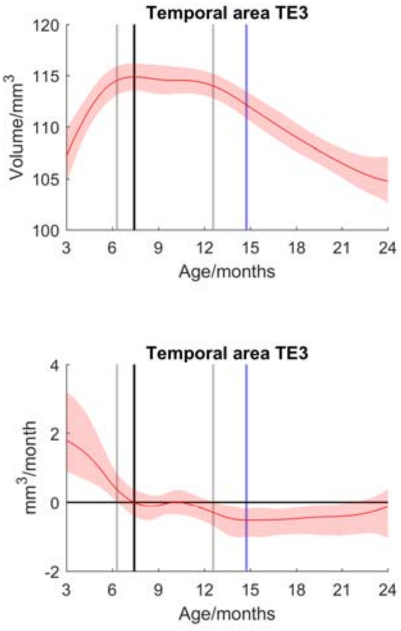

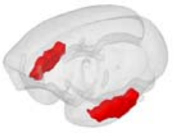

Temporal area TE3
TE3

Peak at 7.42 months [6.30, 12.57]
Maximum rate at 14.74 months.

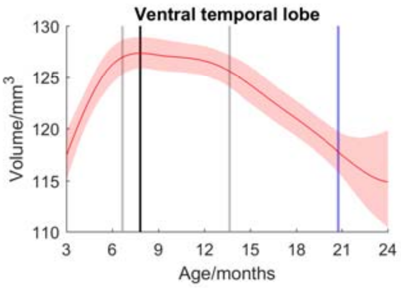

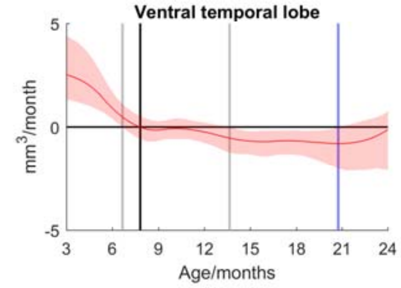

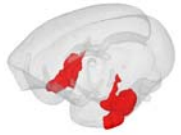

Ventral temporal lobe
TF

Peak at 7.81 months [6.66, 13.64]
Maximum rate at 20.76 months.

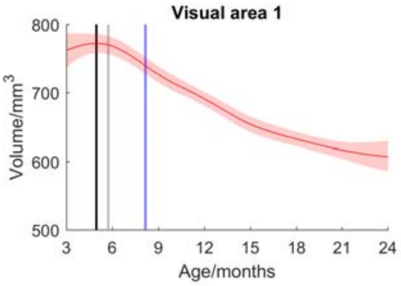

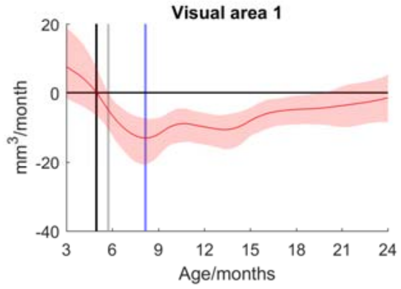

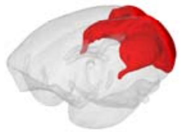

Visual area 1
V1

Peak at 4.95 months [2.51, 5.73]
Maximum rate at 8.15 months.

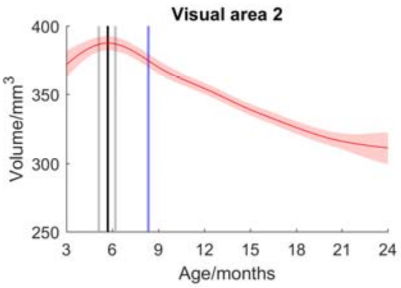

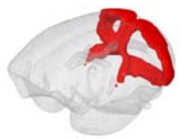

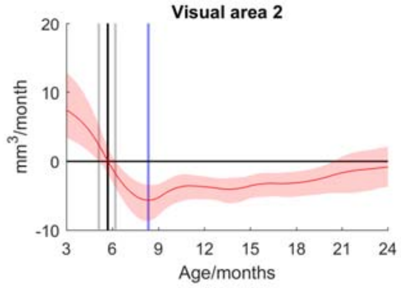

Visual area 2
V2

Peak at 5.70 months [5.12, 6.19]
Maximum rate at 8.34 months.

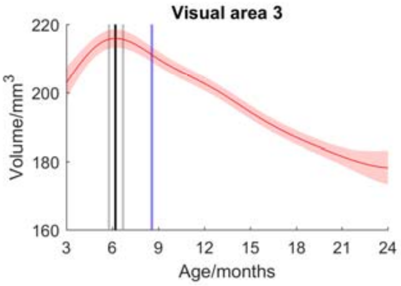

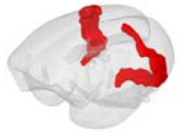

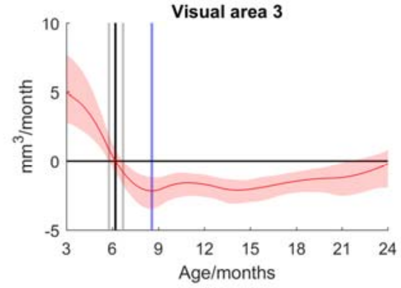

Visual area 3
V3

Peak at 6.19 months [5.78, 6.69]
Maximum rate at 8.57 months.

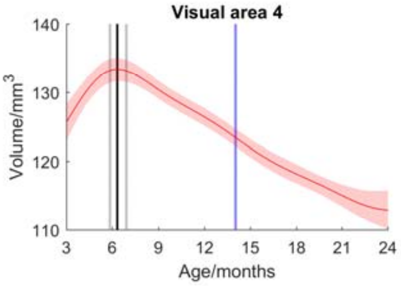

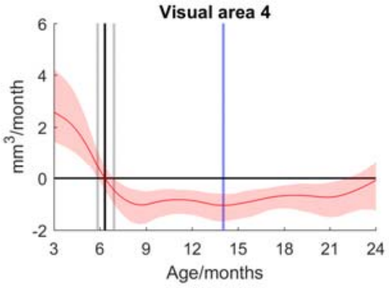

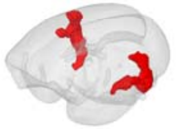

Visual area 4
V4

Peak at 6.32 months [5.84, 6.91]
Maximum rate at 14.03 months.

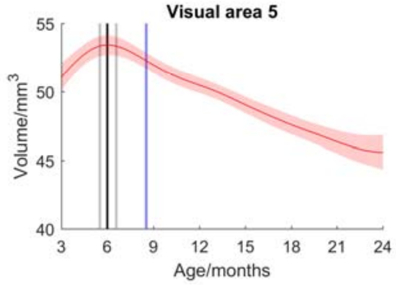

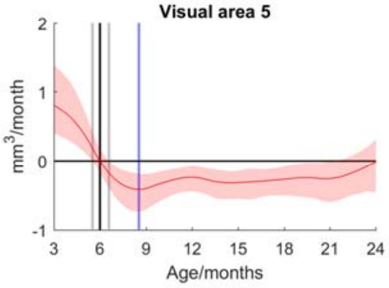

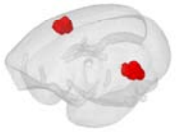

Visual area 5
V5

Peak at 5.99 months [5.51, 6.58]
Maximum rate at 8.53 months.

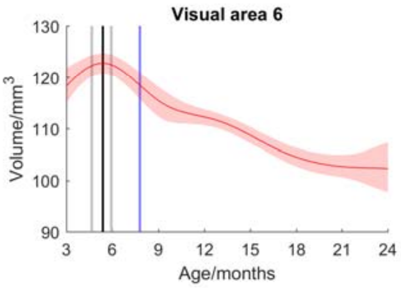

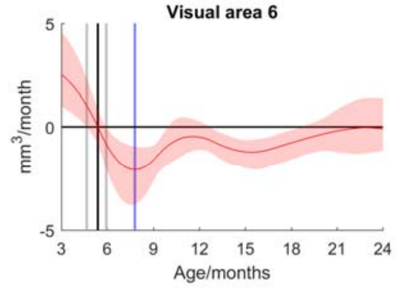

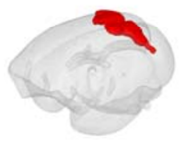

Visual area 6
V6

Peak at 5.38 months [4.65, 5.92]
Maximum rate at 7.79 months.

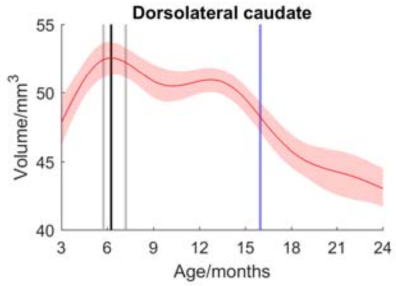

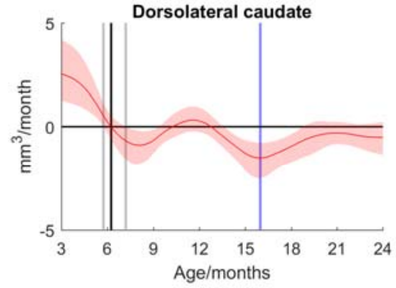

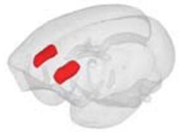

Dorsolateral caudate
DLCaud

Peak at 6.24 months [5.74, 7.19]
Maximum rate at 15.97 months.

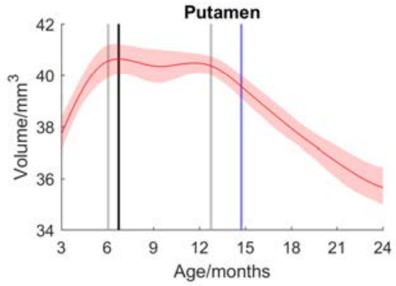

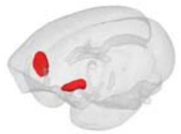

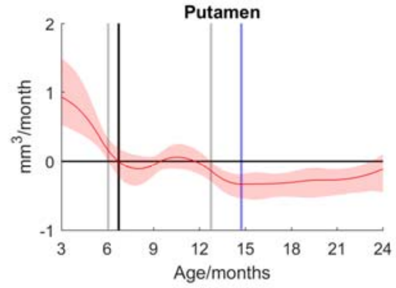

Putamen
Put

Peak at 6.74 months [6.05, 12.76]
Maximum rate at 14.74 months.

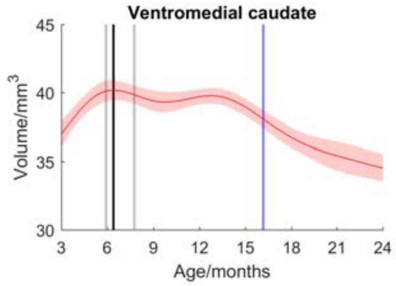

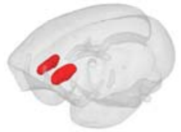

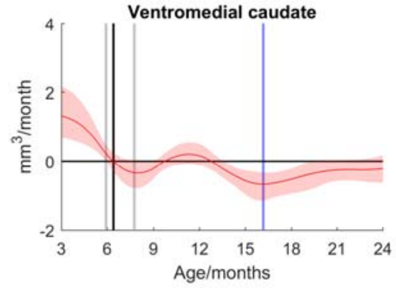

Ventromedial caudate
VMCaud

Peak at 6.39 months [5.91, 7.76]
Maximum rate at 16.17 months.

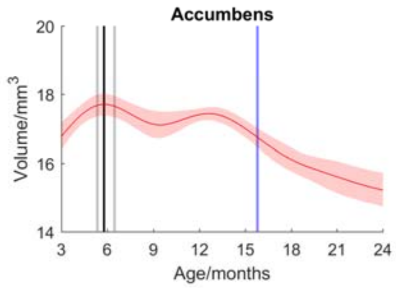

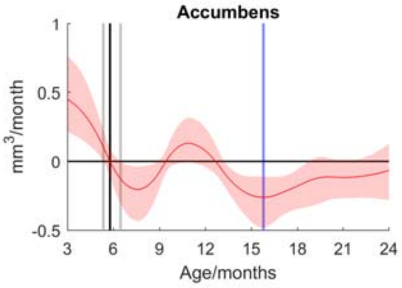

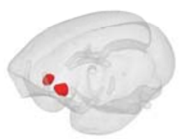

Accumbens
Acb

Peak at 5.78 months [5.34, 6.46]
Maximum rate at 15.79 months.

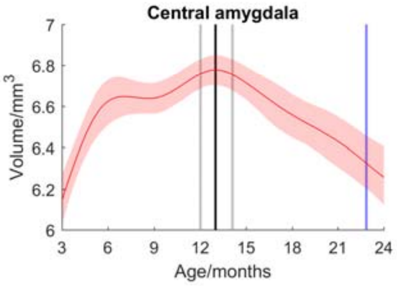

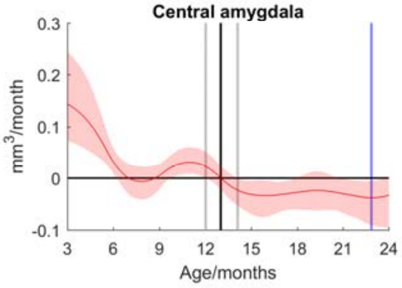

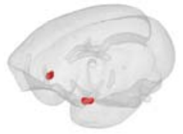

Central amygdala
AmygCe

Peak at 13.00 months [12.02, 14.10]
Maximum rate at 22.86 months.

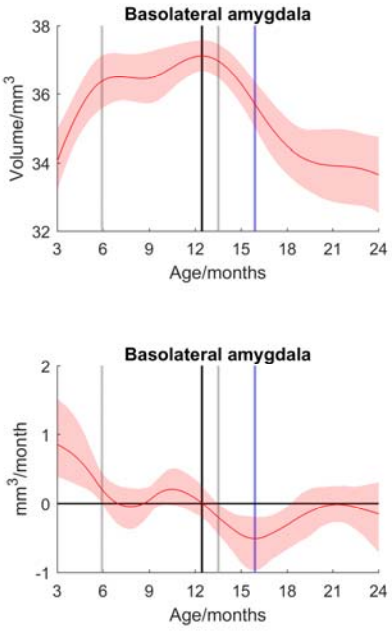

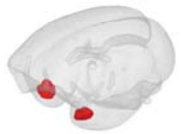

Basolateral amygdala
AmygBL

Peak at 12.44 months [5.92, 13.51]
Maximum rate at 15.90 months.

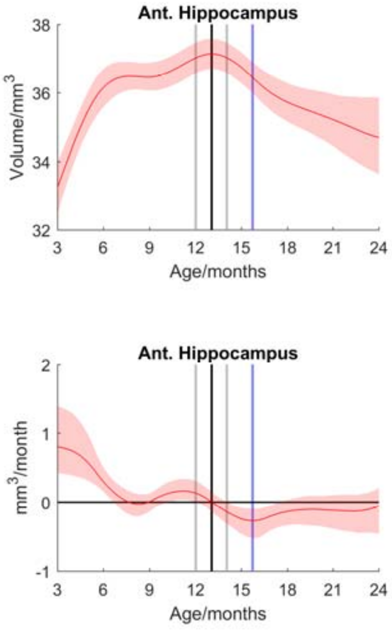

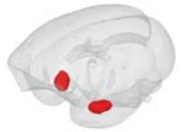

Ant. Hippocampus
antHIPP

Peak at 13.06 months [12.02, 14.05]
Maximum rate at 15.73 months.

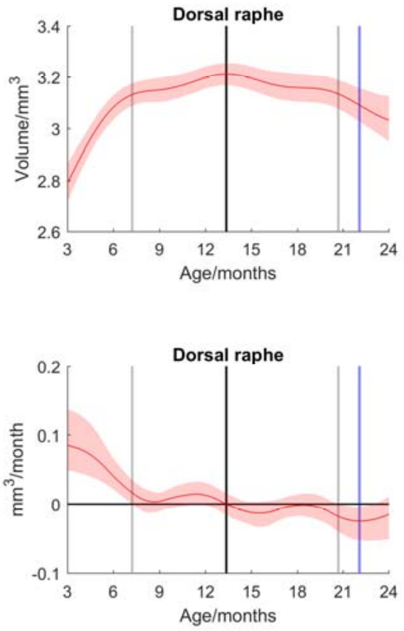

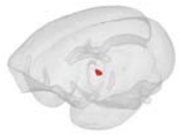

Dorsal raphe
dorsal raphe

Peak at 13.36 months [7.22, 20.70]
Maximum rate at 22.07 months.

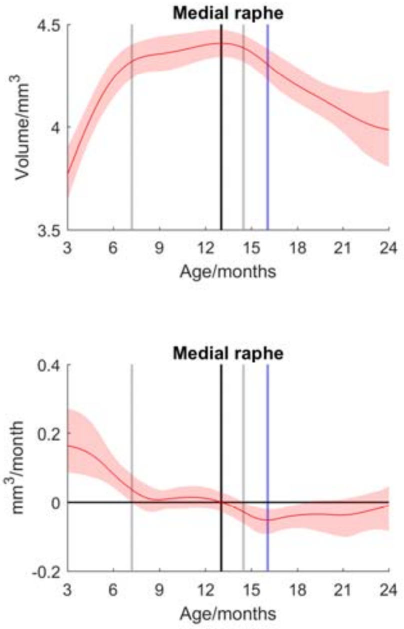

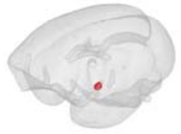

Medial raphe
MRaphe

Peak at 13.03 months [7.20, 14.49]
Maximum rate at 16.06 months.

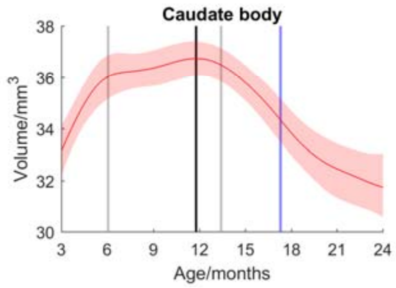

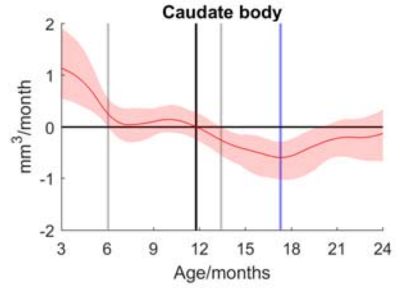

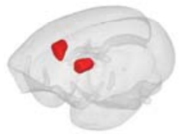

Caudate body
CaudBody

Peak at 11.78 months [6.04, 13.42]
Maximum rate at 17.29 months.

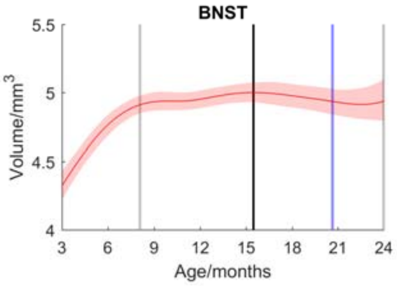

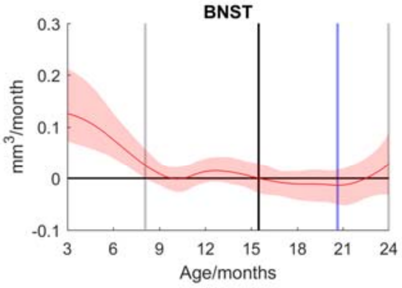

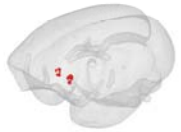

BNST
BNST

Peak at 15.47 months [8.08, 23.98]
Maximum rate at 20.65 months.

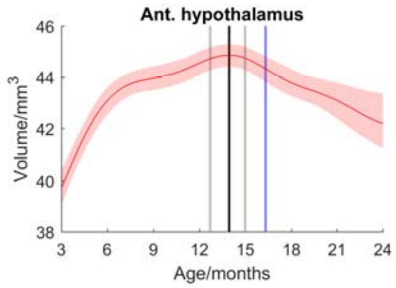

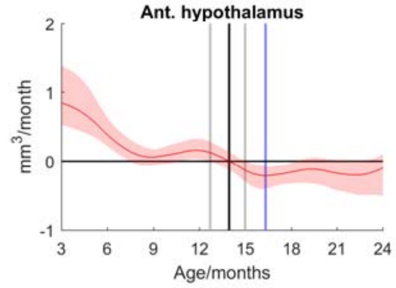

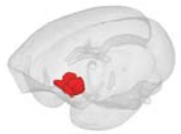

Ant. hypothalamus
antHypo

Peak at 13.94 months [12.70, 14.98]
Maximum rate at 16.32 months.

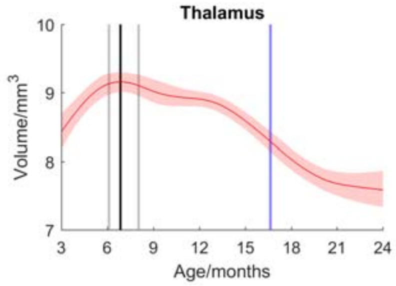

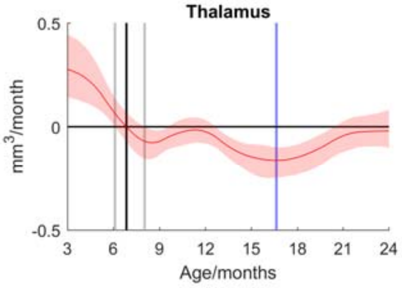

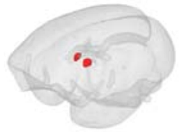

Thalamus
MD Thal

Peak at 6.85 months [6.10, 8.04]
Maximum rate at 16.63 months.

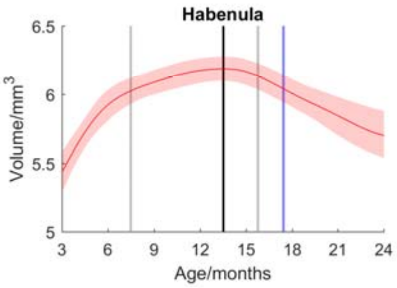

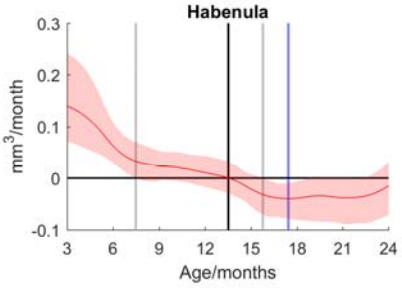

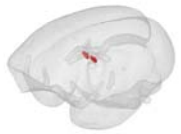

Habenula
Hab

Peak at 13.51 months [7.48, 15.76]
Maximum rate at 17.43 months.

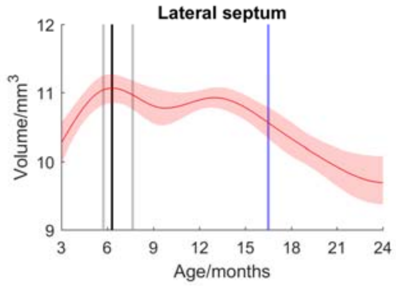

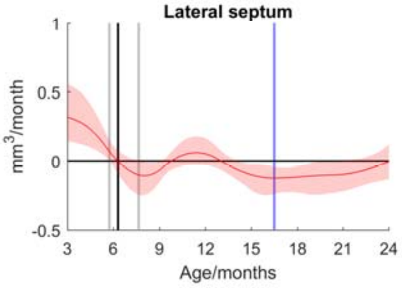

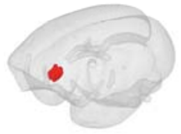

Lateral septum
LSeptum

Peak at 6.30 months [5.74, 7.65]
Maximum rate at 16.49 months.

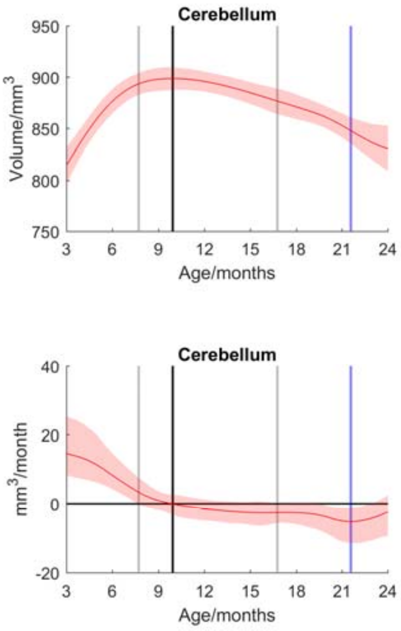

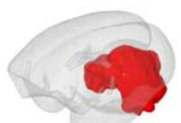

Cerebellum
Cereb

Peak at 9.93 months [7.71, 16.76]
Maximum rate at 21.58 months.
